# Ultraviolet B radiation improves salt-induced responses in the facultative halophyte *Chenopodium quinoa*

**DOI:** 10.1093/plphys/kiaf569

**Published:** 2025-11-10

**Authors:** Giorgia Guardigli, Francesca Alderotti, Ilaria Colzi, Cristina Gonnelli, Stefano Mancuso, Mauro Centritto, Filippo Micheletti, Giovanni Agati, Federico Vita, Hao Zhou, Ian C Dodd, Cecilia Brunetti, Nadia Bazihizina

**Affiliations:** Department of Biology, University of Florence, Florence 50121, Italy; Department of Agriculture, Food, Environment and Forestry, University of Florence, Sesto Fiorentino, Florence 50019, Italy; National Research Council of Italy, Institute for Sustainable Plant Protection (CNR-IPSP), Sesto Fiorentino, Florence 50019, Italy; Department of Biology, University of Florence, Florence 50121, Italy; Department of Biology, University of Florence, Florence 50121, Italy; Department of Agriculture, Food, Environment and Forestry, University of Florence, Sesto Fiorentino, Florence 50019, Italy; National Research Council of Italy, Institute for Sustainable Plant Protection (CNR-IPSP), Sesto Fiorentino, Florence 50019, Italy; National Research Council of Italy, Institute of Applied Physics “Nello Carrara” (CNR-IFAC), Sesto Fiorentino, Florence 50019, Italy; National Research Council of Italy, Institute of Applied Physics “Nello Carrara” (CNR-IFAC), Sesto Fiorentino, Florence 50019, Italy; Department of Bioscience, Biotechnology and Environment, University of Bari "Aldo Moro", Bari 70125, Italy; Lancaster Environment Centre, Lancaster University, Lancaster LA1 4YQ, UK; Lancaster Environment Centre, Lancaster University, Lancaster LA1 4YQ, UK; Department of Agriculture, Food, Environment and Forestry, University of Florence, Sesto Fiorentino, Florence 50019, Italy; National Research Council of Italy, Institute for Sustainable Plant Protection (CNR-IPSP), Sesto Fiorentino, Florence 50019, Italy; Department of Biology, University of Florence, Florence 50121, Italy

## Abstract

In natural environments, plants are continuously exposed to multiple abiotic stresses, such as high salinity and excess ultraviolet (UV)-B radiation. While responses to individual stresses are well understood, less is known about their combined impact. Here, we treated quinoa (*Chenopodium quinoa*) seedlings with salt (0 and 200 mm NaCl) under either photosynthetically active radiation (PAR) or PAR supplemented with UV-B radiation (313 nm, 1 h/d, 1.71 W/m^2^) to investigate their response to combined salt and UV-B stress. While salinity had minimal effects on plant growth, it decreased stomatal conductance and photochemical efficiency by 36% to 47%. UV-B supplementation mitigated the negative effects of salinity, enhancing photosynthetic efficiency and water relations in UV-B- and salt-treated plants. Enhanced leaf water relations in the combined treatment were associated with altered ion translocation and shoot compartmentalization, especially for K^+^. Indeed, UV-B decreased K^+^ accumulation in epidermal bladder cells, suggesting a redistribution from epidermal bladder cells to other leaf tissues. UV-B treatment shifted plant metabolism toward producing hydroxycinnamic acid, while quercetin levels remained unchanged, indicating minimal stress. This study describes a protective mechanism in quinoa where UV-B radiation enhances ion translocation, water relations, and metabolic adjustments, mitigating salinity stress. Our findings offer key insights into plant resilience and physiological adaptation in salt-affected environments under elevated UV-B exposure.

## Teaser

Ultraviolet-B light enhances quinoa adaptation to salt stress by altering foliar metabolism, water management, and ion distribution to epidermal bladder cells.

## Introduction

The growing global population and ongoing climate change pose critical challenges to agriculture. While population growth increases the demand for food, climate change intensifies the environmental stresses affecting crop productivity (Godfray et al. 2010; Ray et al. 2013; Suzuki et al. 2014; Pereira 2016; Malhi et al. 2021). Emerging evidence indicates that the combined impact of multiple stresses can lead to unexpected outcomes that are not predictable by studying single stresses separately (Mittler 2006; Suzuki et al. 2014; Pascual et al. 2022; Zandalinas and Mittler 2022). Soil salinization is a widespread environmental challenge that affects *circa* 10% of the worlds’ land area (FAO 2024). This issue often co-occurs with elevated ultraviolet (UV-B) radiation, a significant environmental stressor driven by stratospheric ozone depletion (Barnes et al. 2019). Climate change models predict that both UV-B irradiance and salinity will concurrently increase in many regions worldwide (Barnes et al. 2019; Corwin 2021; Hassani et al. 2021; Barnes et al. 2022). Although plant responses to either salinity or UV-B as individual stressors are well-documented, their combined effects are less studied. Salinity negatively affects plant growth primarily through: (i) disrupting water relations because of osmotic stress; (ii) direct cellular damage caused by ion toxicities (mainly sodium (Na^+^) and chloride (Cl^−^)) and nutrient imbalances (e.g, potassium (K^+^) deficiency)), and (iii) oxidative damage induced by excessive reactive oxygen species (ROS) production (Shabala and Pottosin 2014; van Zelm et al. 2020; Melino and Tester 2023). Conversely, UV-B radiation functions both as a regulatory signal and a stressor according to the dose. Although negative synergistic interactions between stresses can exacerbate plant stress (Zlatev et al. 2012; Ma et al. 2016), combined salt and UV-B exposure can have antagonistic effects, with less severe impact than the sum of their individual effects (Ouhibi et al. 2014; Ma et al. 2016; Mohamed et al. 2023). At the same time, negative synergistic interactions that exacerbate plant stress responses have also been reported (Zandalinas and Mittler 2022; Fitzner et al. 2023). Despite these contrasting results, a critical aspect that so far remains unexplored is the impact of UV-B radiation on ion relations. Since radiation quality, such as red and blue light, modulates root ion uptake and translocation (Mankotia et al. 2024), and ion regulation is crucial for plants to survive under saline conditions, it is important to investigate whether UV-B radiation affects ion (particularly K^+^, Na^+^, and Cl^−^) relations in salt-treated plants.

Halophytes have evolved to thrive in extreme and inhospitable environments where multiple stress factors, such as high UV-B radiation and drought, co-occur with salinity (Nikalje et al. 2019; Lopes et al. 2023). They represent promising candidates to understand the mechanisms underpinning cross-tolerance to multiple stresses (Hamed et al. 2013; Shabala 2013; Nikalje et al. 2019). Among halophytes, quinoa (*Chenopodium quinoa* Willd.), a tetraploid annual pseudocereal crop, has attracted significant attention as it can adapt to diverse environmental conditions and has high nutritional value (Angeli et al. 2020). As a facultative halophyte, quinoa has a good tolerance to salinity, with optimal growth around 100 mm NaCl (Hariadi et al. 2011). Its salt tolerance mechanisms are primarily associated with efficient Na^+^ exclusion and enhanced regulation of tissue-specific and ROS-specific K^+^ retention in roots (Cai and Gao 2020; Bazihizina et al. 2022; Tanveer et al. 2024). Additionally, quinoa seems tolerant to elevated UV-B radiation (e. g. 7.5 W/m^2^), likely due to constitutive traits such as stable pigment composition, accumulation of UV-screening compounds, and anatomical adaptations such as epidermal bladder cells (EBCs) (González et al. 2009; Perez et al. 2015).

Halophytes achieve salt tolerance by coordinating various physiological, anatomical, and morphological traits. One of the most striking adaptations contributing to salt tolerance in many halophytes, including quinoa, is the ability to secrete salt out of leaf tissues through EBCs and salt glands (Supplementary Fig. S1). This mechanism is considered a critical determinant of salt tolerance. Although removing quinoa EBCs impairs responses to high salinities by decreasing growth, disrupting ion homeostasis, and altering levels of key osmolytes and metabolites (Kiani-Pouya et al. 2017), the precise role of EBCs in salt tolerance remains unclear and is still a subject of debate (Moog et al. 2022). They are proposed to store metabolites and act as external reservoirs for water and/or ROS scavenging compounds and organic osmoprotectants (Hasegawa et al. 2000; Agarie et al. 2007; Kiani-Pouya et al. 2017, 2019, 2020; Bazihizina et al. 2022). Additionally, EBCs have also been identified as crucial for protecting leaves against UV-B radiation damage, acting as a secondary epidermal layer that provides physical shielding and serves as reservoirs for UV-screening metabolites and ROS-scavenging compounds (Kiani-Pouya et al. 2017; Imamura et al. 2020). Alternatively, bladder cells may function as an ABA-producing factory (Zou et al. 2017), thus playing a pivotal role in mediating plant responses to combined abiotic stresses such as salinity and UV-B radiation. Although the functional significance of EBCs in plants exposed to concurrent UV-B and salinity has not been considered, they could contribute to the maintenance of ion homeostasis and osmotic balance under stress conditions via ABA-regulated stomatal closure or by altering ion compartmentation in the leaves.

In this study, we examined the physiological responses of quinoa seedlings to salinity in combination with high UV-B radiation, with a particular focus on how these concurrent stresses affect water and ion relations. Since Na^+^ and K^+^ dynamics are critical in salt-treated plants and radiation quality may influence ion uptake and translocation, potentially via modulation of transcription factors (Mankotia et al. 2024), we hypothesized that UV-B radiation could modify K^+^ and Na^+^ homeostasis and compartmentalization within salt-stressed leaves and EBCs, thereby affecting overall plant performance under saline conditions.

## Results

### Plant growth

**Figure 1.**
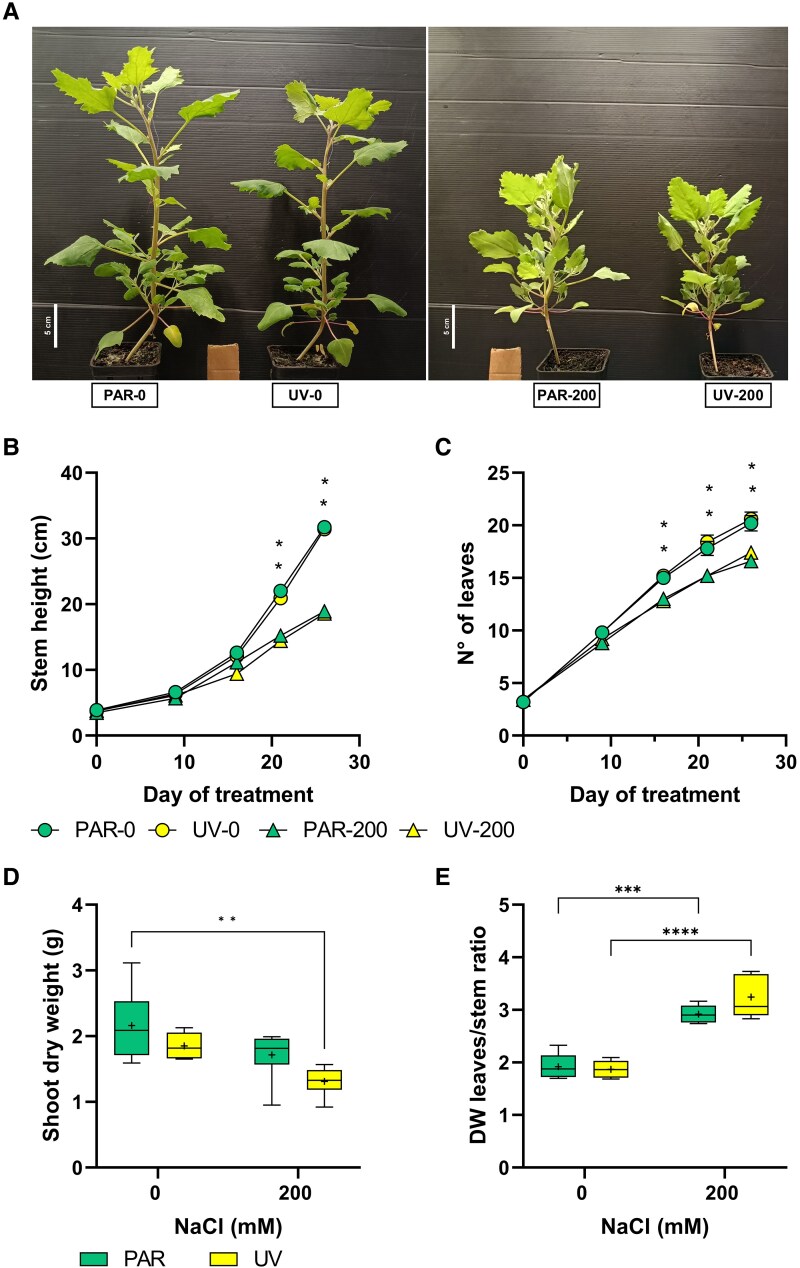
Growth of quinoa under 4 treatments over 26 d: PAR with tap water (PAR-0), PAR with 200 mm saline water (PAR-200), supplemental UV-B radiation with tap water (UV-0), and supplemental UV-B radiation with 200 mm saline water. **A)** Visible effects of treatments on representative plants from each group. **B)** Stem height, **C)** number of leaves on the primary stem, **D)** shoot dry weight, and **E)** dry weight (DW) leaves/stem ratio. In **B** and **C**, data are presented as means ± SE (*n* = 5). Asterisks (* *P* ≤ 0.05, ** *P* ≤ 0.01, *** *P* ≤ 0.001, **** *P* ≤ 0.0001) indicate significant differences based on a 2-way ANOVA followed by Tukey's multiple comparison test. In **D** and **E**, the top and bottom of each box represent the 25th and 75th percentiles, respectively. The horizontal line inside each box represents the median, the “+” symbol indicates the mean (*n* = 5), and the whiskers show the minimum and maximum values.

While leaf elongation measurements did not differ between treatments (Supplementary Fig. S2), salt addition decreased stem height and leaf number by 40% and 17%, respectively, compared to the relative controls (Fig. 1, A to C). UV-B treatment did not affect these variables. Shoot dry weight was similar across the 4 treatments (Fig. 1D). However, salt treatment increased biomass allocation to leaves and decreased allocation to stems and roots (Supplementary Table S1). Consequently, leaf/stem dry weight ratio increased by 1.5-fold and 1.7-fold in photosynthetically active radiation (PAR)-200 and UV-200, respectively, compared to their controls (Fig. 1E). Similarly, the shoot/root ratio in PAR-200 increased by 1.6-fold and in UV-200 by 1.3-fold compared to the relative controls. The UV treatment did not significantly affect the leaves/stem ratio. Salinity only decreased root dry weight by 44% in the PAR-200 treatment (Supplementary Table S1). Under control conditions, UV-B exposure also increased the specific leaf area (SLA), with changes significant only when compared with UV-200 (Supplementary Table S1). By contrast, in all other treatments, SLA values remained within the 440 to 470 cm² g⁻¹ range.

### Water relations

**Figure 2.**
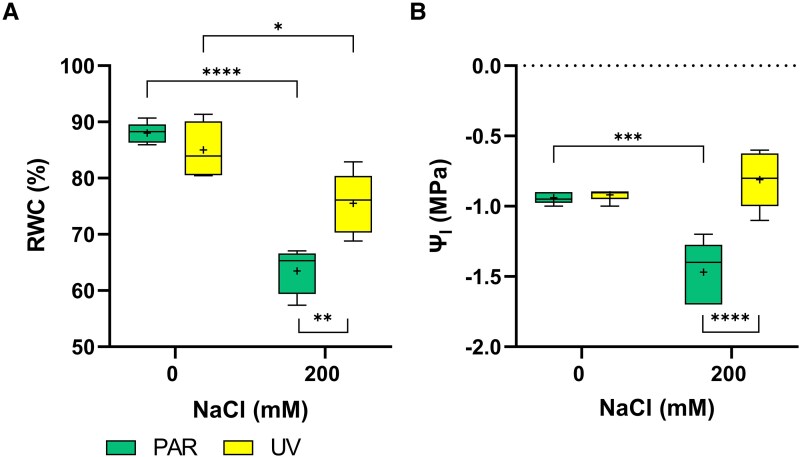
Water relations under the 4 treatments. **A)** Leaf relative water content (RWC; %) and **B)** leaf water potential (Ψ_l_, MPa). A 2-way ANOVA followed by Tukey's multiple comparisons test was conducted to assess significant differences (* *P* ≤ 0.05, ** *P* ≤ 0.01, *** *P* ≤ 0.001, **** *P* ≤ 0.0001). Top and bottom of each box represent the 25th and 75th percentiles, the horizontal line inside each box represents the median, the «+» inside each box represents the average (*n* = 5), and the whiskers represent the minimum and maximum values.

Compared to controls, salt treatment significantly decreased relative water content (RWC) by 28% in the PAR treatment but by 11% in the UV treatment (Fig. 2A), resulting in a leaf RWC in UV-200 plants 1.2 times higher than in PAR-200 plants. Similar changes were also observed for Ψ_l_, with this value decreasing only in the PAR-200 treatment (Fig. 2B).

### Chlorophyll fluorescence

**Figure 3.**
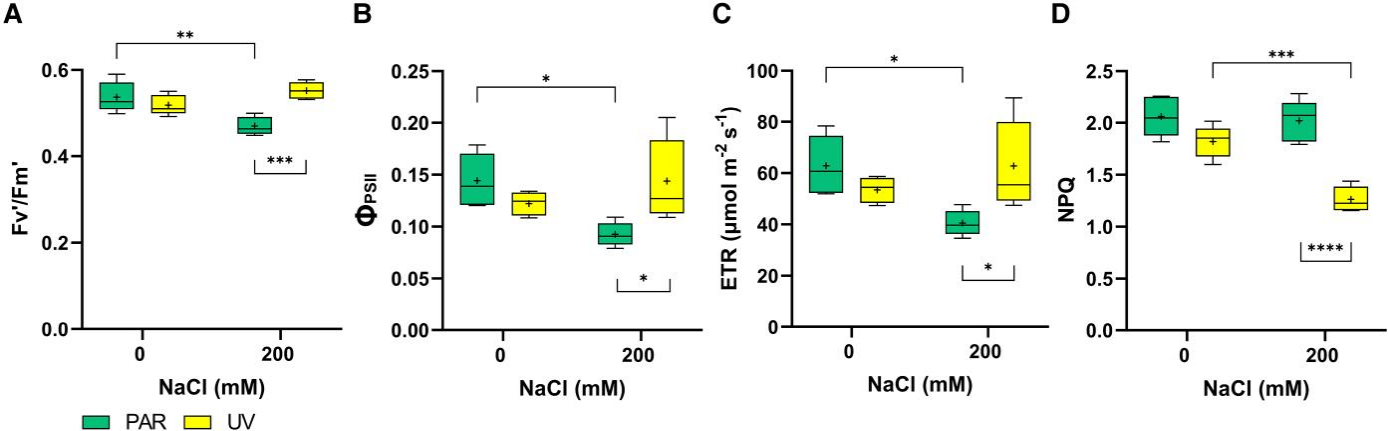
Responses of the chlorophyll fluorescence parameters to the 4 treatments. Measurements were made on the youngest fully expanded leaves on Day 26. **A)**  *F*_v_’/*F*_m_’ (capture efficiency of excitation energy by the open, oxidized PSII reaction center in the light), **B)** Φ_PSII_ (PSII efficiency in light-adapted leaves), **C)** ETR (electron transport rate), and **D)** NPQ (nonphotochemical quenching). All treatments showed an average *F*_v_/*F*_m_ of 0.81 (not shown in the figure). A 2-way ANOVA followed by Tukey's multiple comparisons test was performed. The graph shows significant differences (*P ≤ 0.05, **P ≤ 0.01, ***P ≤ 0.001, ****P ≤ 0.0001). The top and bottom of each box represent the 25th and 75th percentiles, the horizontal line within each box represents the median, the “+” symbol indicates the average (*n* = 5), and the whiskers show the minimum and maximum values.

Neither salinity nor UV exposure significantly affected *F*_v_/*F*_m_ (Supplementary Table S2). On the other hand, salinity differentially affected chlorophyll fluorescence parameters in light-adapted leaves in UV and PAR plants. In PAR plants, adding 200 mm NaCl decreased *F*_v_’/*F*_m_’ by 13% (Fig. 3). By contrast, no salt-induced reduction occurred in UV-treated plants, where *F*_v_’/*F*_m_’ values were comparable to those in PAR-0 and UV-0 plants and 15% higher than those in PAR-200 plants (Fig. 3A). Similarly, Φ_PSII_ and electron transport rate (ETR) decreased (36%) only in PAR-200 plants (Fig. 3B and C). Finally, nonphotochemical quenching (NPQ) significantly decreased in UV-200 plants, decreasing by 31% compared to UV-0 and by 38% compared to PAR-200 (Fig. 3D). Photosynthetic performance aligned with chlorophyll fluorescence data, with more pronounced *P*_n_ declines in PAR-200 plants. Indeed, salt treatment decreased *P*_n_ by 61% under PAR treatment but by only 38% under UV treatment (Supplementary Fig. S3A). Stomatal conductance showed salt-induced reductions in the PAR treatment (Supplementary Fig. S3B). In contrast, UV treatment alone did not significantly affect *P*_n_ or *g*_s_ when compared to PAR-0 plants.

### Pigment concentration

**Figure 4.**
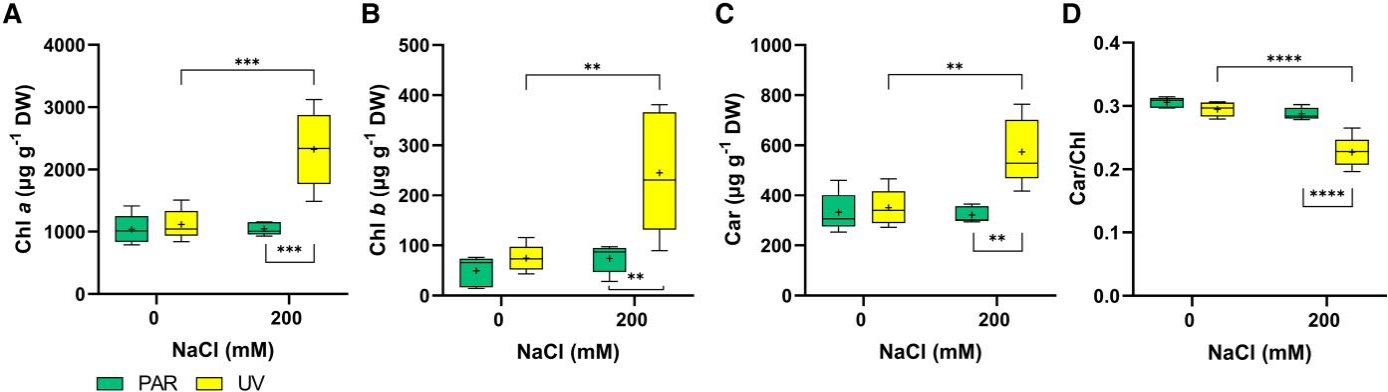
Pigment concentration in the youngest fully expanded leaves under the 4 treatments. **A)** chlorophyll *a* (chl *a*) concentration, **B)** chlorophyll *b* (chl *b*) concentration, **C)** carotenoids (car) concentration, **D)** ratio of carotenoids to total chlorophyll (car/chl). A 2-way ANOVA followed by Tukey's multiple comparisons test was performed. The graph shows significant differences (*P ≤ 0.05, **P ≤ 0.01, ***P ≤ 0.001, ****P ≤ 0.0001). The top and bottom of each box represent the 25th and 75th percentiles, the horizontal line inside each box represents the median, the «+» inside each box represents the average (*n* = 5), and the whiskers represent the minimum and maximum values.

The combined salt and UV treatment affected chlorophyll (chl) *a*, chl*b*, and carotenoid concentrations. Chl*a* and chl*b* concentrations increase in UV-200 plants by 2.1- and 3.3-fold, respectively, compared to the other treatments (Fig. 4, A and B). Despite a significant increase in carotenoid concentration (1.6-fold, Fig. 4C), in UV-200 plants, the car/chl*a*  *+*  *b* ratio declined by 23% (Fig. 4D).

### Tissue ion concentrations

Ion concentrations were measured in intact (i.e. nonbrushed) young leaves and youngest fully expanded leaves, and stems (Table 1). Salt stress increased K^+^ concentrations of young leaves by 1.5- and 1.2-fold in the PAR and UV treatments, respectively. Without salt, UV treatments increased leaf K^+^ concentrations of the youngest fully expanded leaves by 1.2-fold. Salt-treated plants further increased leaf K^+^ concentrations by 1.6-fold and 1.3-fold in PAR-200 and UV-200 plants, respectively.

**Table 1.** Concentration of K^+^, Na^+^, and Cl^−^ in the different plant tissues: young leaves (YL), youngest fully expanded leaves (YFEL), and stem

	K^+^ concentrations (mM)				Na^+^ concentrations (mM)				Cl^−^ concentrations (mM)			
YL	285.5 ± 13.8b	418.5 ± 14.9a	313.4 ± 7.9b	378.6 ± 20.5a	2.0 ± 0.3b	17.3 ± 2.5a	3.5 ± 0.5b	17.0 ± 2.0a	103.9 ± 2.2b	222.6 ± 33.5a	79.7 ± 4.6b	261.5 ± 12.4a
YFEL	267.6 ± 4.2b*	428.2 ± 5.0a	310.0 ± 8.3b*	414.4 ± 13.4a	6.8 ± 1.0b	48.24.0a	8.5 ± 1.0b	62.2 ± 5.8a	34.4 ± 1.7b	164.1 ± 18.4a	30.3 ± 1.9b	157.1 ± 8.0a
Stem	261.4 ± 3.4b	392.9 ± 13.2a	280.2 ± 3.1b	415.5 ± 6.0a	13.9 ± 0.8b	117.3 ± 13.1a*	14.4 ± 1.3b	84.3 3.7a*	80.0 ± 7.2b	234.1 ± 13.0a	86.2 ± 4.4b	260.3 ± 3.3a
	PAR 0	PAR 200	UV 0	UV 200	PAR 0	PAR 200	UV 0	UV 200	PAR 0	PAR 200	UV 0	UV 200

The treatments listed at the bottom of the table include PAR with tap water (PAR-0), PAR with 200 mm saline water (PAR-200), UV-B radiation with tap water (UV-0), and UV-B radiation with 200 mm saline water (UV-200). The table shows significant differences (*P* ≤ 0.05) between salt treatments (PAR-0 vs. PAR-200 and UV-0 vs. UV-200) using lowercase letters and between radiation treatment (PAR-0 vs. UV-0 and PAR-200 vs. UV-200) using an asterisk. Data are presented as means ± SE (*n* = 5).

Leaf Na^+^ concentrations also increased in salt-treated plants under both PAR and UV light, albeit to a much lower extent. While K^+^ values in salt-treated plants always exceeded 300 mm, Na^+^ values ranged between 17 and 117 mm. Nevertheless, values in nonbrushed salt-treated young leaves increased by 8.8- and 4.8-fold, respectively, in PAR-200 and UV-200 compared to the relative controls (Table 1). Likewise, in the youngest fully expanded leaves, Na^+^ concentrations increased by 7.1 to 7.3-fold in PAR-200 and UV-200 compared to the relative controls. Both in young leaves or the youngest fully expanded leaves, there were no significant differences between PAR-0 and UV-0, or between PAR-200 and UV-200. The 3-way ANOVA reveals a significant interaction between the type of tissue, salt, and UV treatment but only for Na^+^ (Supplementary Table S3). Conversely, there was no significant interaction between these factors for Cl^−^ concentration; however, for K^+^, a significant interaction was observed between the UV and salt treatment.

The salt treatment affected the Cl^−^ concentration in both nonbrushed young leaves and the youngest fully expanded leaves. Indeed, in young leaves, salinity in PAR-200 and UV-200 plants, respectively, led to a 2.1- and 3.3-fold increase in leaf Cl^−^ compared to the relative controls. In the youngest fully expanded leaves, values increased by 4.8- and 5.2-fold, respectively (Table 1). No differences in Cl^−^ were found between PAR-0 and UV-0, or between PAR-200 and UV-200, for both young leaves and youngest fully expanded leaves (Table 1).

Compared to controls, salinity increased stem K^+^ concentrations by 1.5-fold in both PAR and UV-treated plants, while stem Na^+^ increased by 8.4-fold and 5.8-fold in PAR- and UV-plants, respectively. Similar salt-induced increases were observed for stem Cl^−^, with values 3-fold greater than the relative controls in both PAR- and UV-treated plants.

Both salt and UV treatments decreased K^+^/Na^+^ ratio in young leaves compared to PAR-0 plants (Supplementary Table S4). In particular, salt treatment decreased this ratio by 83% and 75% in PAR-200 and UV-200 plants compared to their respective controls (PAR-0 and UV-0). By contrast, in the youngest fully expanded leaves, only the salt treatment reduced K^+^/Na^+^ ratio, with a 79% to 82% decrease in both PAR-200 and UV-200 plants.

**Figure 5.**
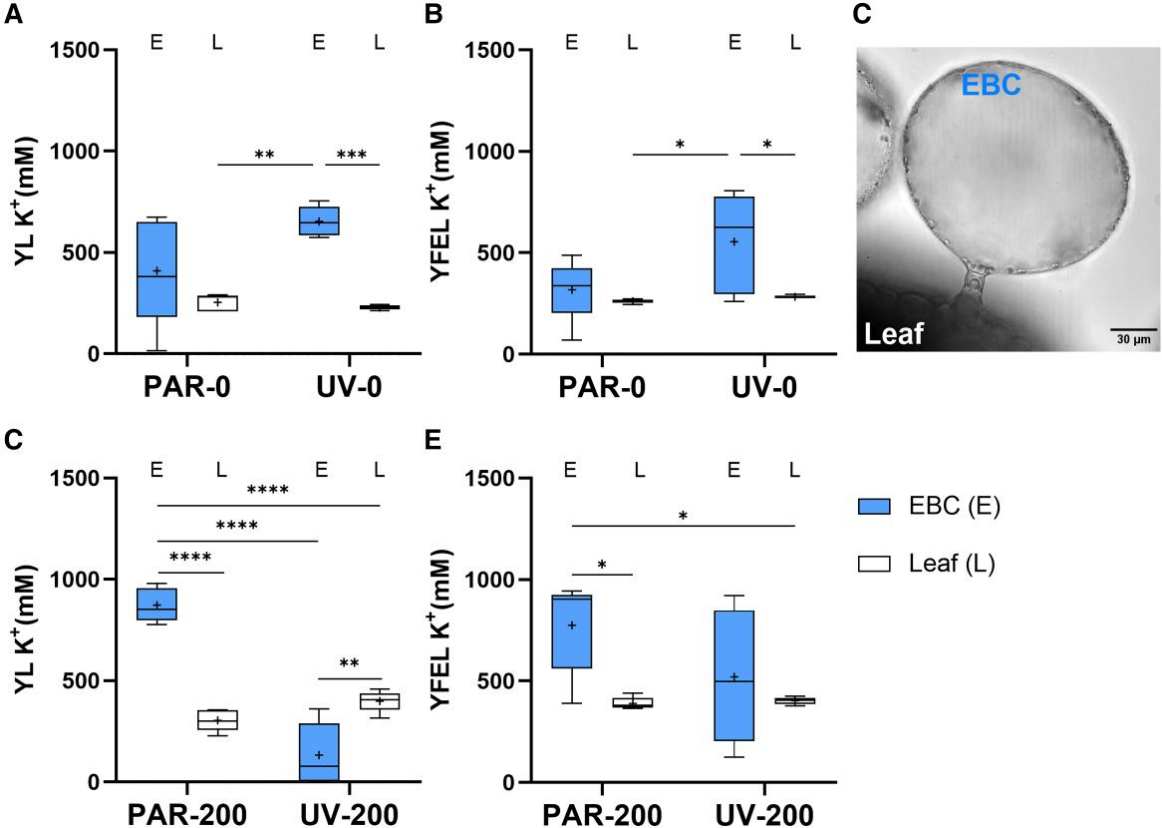
Comparison of K^+^ concentration between EBCs and leaf tissue in both young leaves (YL) and youngest fully expanded leaves (YFEL). K^+^ concentration in YL **A)** and YFEL **B)** in plants irrigated with tap water. **C)** Microscopic view of EBCs from a control plant (image reused from Supplementary Fig. S1C). K^+^ concentration in YL **D)** and YFEL **E)** in plants treated with salt. A 2-way ANOVA followed by Tukey's multiple comparisons test was performed, and the graph shows only significant differences (*P ≤ 0.05, **P ≤ 0.01, ***P ≤ 0.001, ****P ≤ 0.0001) between EBCs and leaf tissue. The *x*-axis of the boxplot represents the light and salt treatments, with the top and bottom of each box representing the 25th and 75th percentiles. The horizontal line inside each box indicates the median, the “+” symbol represents the average (*n* = 5), and the whiskers show the minimum and maximum values.

As quinoa uses EBCs to sequester ions (Bazihizina et al. 2022), K^+^, Na^+^, and Cl^−^ concentrations between intact/nonbrushed (i.e. leaf including bladders) and brushed (i.e. the leaf without the bladders) leaves (*cf.* Kiani-Pouya et al. 2017; Bazihizina et al. 2022) were compared to estimate ion concentrations with and without EBCs. While Na^+^ and Cl^−^ concentrations did not significantly differ between nonbrushed and brushed leaves in both young and youngest fully expanded leaves (Supplementary Table S5), this comparison highlighted different patterns in K^+^ compartmentalization between the EBCs and the leaf tissues (i.e. with no EBCs, Fig. 5). Indeed, while no significant differences were observed in PAR-0, in UV-0 estimated K^+^ concentrations were always greater in EBCs compared to the brushed leaf tissues (Fig. 5, A and B). In salt-treated plants, 2 contrasting accumulation patterns emerged. Indeed, in PAR-200 plants, the estimated K^+^ concentration in EBCs was 2.9- and 2.0-fold greater than the values in brushed leaves, in young leaves, and in the youngest fully expended leaves, respectively. By contrast, in UV-200, K^+^ concentrations in EBCs dramatically declined, with no significant difference found between EBCs and leaf concentration in the youngest fully expanded leaves, and a 19% decline compared to concentrations in the brushed leaves in young leaves.

Salt treatment almost doubled the ion contribution to leaf OP in both PAR-200 and UV-200 plants. Among the inorganic solutes, K^+^ was the major contributor, accounting for 62% to 77% of the total leaf OP across all treatments (Table 2). The OP attributed to K^+^ was significantly affected by the salt treatment, with a 1.6-fold increase in PAR-200 compared to PAR-0 and a 1.3-fold increase in UV-200 compared to UV-0. Additionally, UV-0 plants showed a 1.2-fold higher K^+^ OP than in PAR-0 plants. While UV radiation treatment alone did not affect Na^+^ OP, salinity increased Na^+^ OP by 12-fold in PAR-200 and by 7-fold in UV-200 compared to the relative controls. Finally, as for K^+^ and Na^+^, the OP due to Cl^−^ was significantly affected by the salt treatment, with a 4.8-fold increase in PAR-200 and a 5.4-fold increase in UV-200 compared to relative controls.

**Table 2.** Osmotic potential of solutes (Ψ_S_) and their percentage contributions in leaf tissues

Contribution of solutes	PAR-0		UV-0		PAR-200		UV-200	
	MPa	%	MPa	%	MPa	%	MPa	%
K^+^	−0.66 b*	72.0	−0.78 b*	77.4	−1.07 a	61.7	−1.02 a	64.1
Na^+^	−0.01 b	1.3	−0.02 b	2.2	−0.12 a	6.0	−0.14 a	8.4
Cl^−^	−0.08 b	8.4	−0.07 b	7.8	−0.38 a	21.9	−0.38 a	23.1
∑ ion	−0.75 b	81.8	−0.88 b	87.3	−1.56 a	89.7	−1.53 a	93.7
Fructose	−0.02 a	1.7	−0.05 a	4.5	−0.05 b	3.1	−0.03 a	1.7
Glucose	−0.03 b	3.8	−0.04 b	4.4	−0.06 a	3.3	−0.04 b	2.6
Sucrose	−0.12 a*	12.7	−0.04 b*	3.8	−0.06 b	3.9	−0.03 b	2
∑ sugar	−0.17 a	18.2	−0.13 a	12.7	−0.18 a*	10.3	−0.10 a*	6.3
Ψs (∑ solutes)	−0.92		−1.01		−1.74		−1.63	

Data are presented as means (*n* = 4). Significant differences (*P* ≤ 0.05, 2-way ANOVA followed by Tukey's test) between salt treatments (PAR-0 vs. PAR-200 and UV-0 vs. UV-200) are shown using lowercase letters, and significant differences between radiation treatments (PAR-0 vs. UV-0 and PAR-200 vs. UV-200) are shown using an asterisk.

While compared to relative controls, salinity did not significantly affect the total sugar OP (Table 2 and Supplementary Table S6). The total sugar OP of PAR-200 was 1.8 times higher than UV-200. When examining individual sugars, the OP of fructose was 2.5-fold higher in PAR-200 compared to PAR-0. By contrast, for glucose OP, there were no significant effects observed due to the UV radiation exposure or the combined salt and UV treatment. In terms of sucrose OP, both salt and UV treatments had significant individual effects. In PAR-200, sucrose OP decreased by 50% compared to PAR-0, and in UV-0, it was reduced by 67% compared to PAR-0.

### Secondary metabolites

From the polyphenols analysis, 12 principal peaks were identified in the youngest fully expanded leaves (Supplementary Table S7). To provide an overview of the plant secondary metabolism, the compounds separated by high-performance liquid chromatography (HPLC) were grouped by classes: hydroxycinnamic acids (sinapic acid and coumaric acid derivatives), quercetin derivatives (rutin and an unidentified quercetin derivative), and kaempferol derivatives (Table 3). Overall, UV treatment shifted the metabolism towards the production of hydroxycinnamic compounds, with concentrations 1.7-fold higher in UV-0 compared to PAR-0 and 1.5-fold higher in UV-200 compared to PAR-200. No significant differences were found in the concentrations of quercetin and kaempferol derivatives.

**Table 3.** Secondary metabolites concentration in the youngest fully expanded leaves of quinoa after 26 d of treatment

Class of compounds (mg/g DW)	PAR-0	UV-0	PAR-200	UV-200
Hydroxycinnamic acids	9.8 ± 1.6 *	16.7 ± 0.5 *	9.3 ± 0.3 *	13.9 ± 0.9 *
Quercetin derivatives	8.6 ± 1.3	7.3 ± 1.0	5.5 ± 1.2	6.5 ± 0.5
Kaempferol derivatives	0.4 ± 0.1	0.8 ± 0.2	1.4 ± 0.4	0.9 ± 0.3

Data are presented as means ± SE (*n* = 5). The table shows significant differences (*P* ≤ 0.05, 2-way ANOVA followed by Tukey's test) between salt treatments (PAR-0 vs. PAR-200 and UV-0 vs. UV-200) using lowercase letters, and between radiation treatments (PAR-0 vs. UV-0 and PAR-200 vs. UV-200) using an asterisk.

### Gene expression analysis

**Figure 6.**
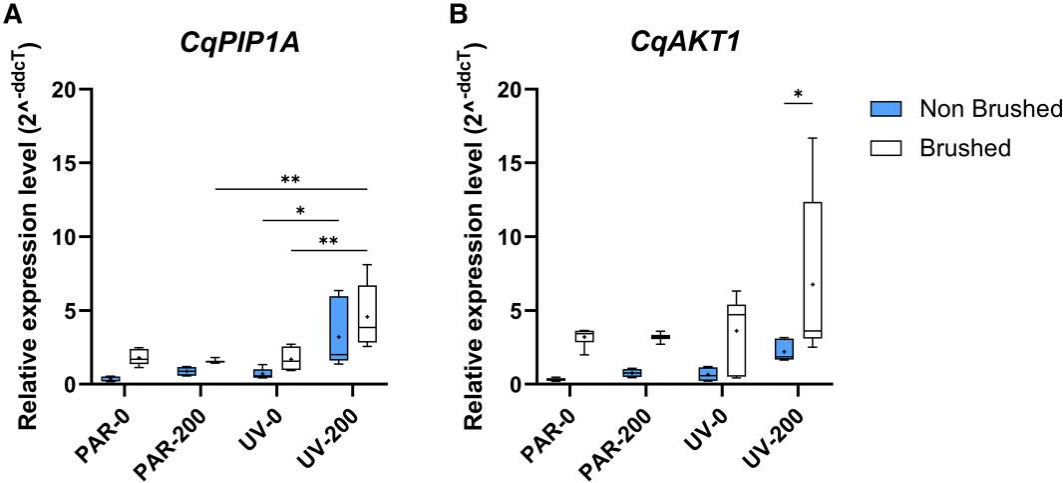
Gene expression patterns in nonbrushed (i.e. intact) and brushed young leaves of quinoa after 26 d of treatment. **A)** PIP1A, plasma membrane aquaporin and **B)** AKT1, voltage-gated K^+^ channel. Two-way ANOVA with Tukey's multiple comparisons test was performed, and only differences between EBCs and leaf tissues and between treatments within the same tissue (brushed or nonbrushed) are shown (*P ≤ 0.05, **P ≤ 0.01, ***P ≤ 0.001, ****P ≤ 0.0001). The *x*-axis of the boxplot represents the light and salt treatment. The top and bottom of each box represent the 25th and 75th percentiles, respectively. The horizontal line inside each box represents the median, the «+» inside each box represents the average (*n* = 3), and the whiskers represent the minimum and maximum values.

The gene expression mainly highlighted an UV-dependent changes in the expression levels of plasma membrane aquaporin (*PIP1A*) in salt-treated plants, with a 3-fold increase in brushed UV-200 leaves compared to brushed PAR-200 leaves (Fig. 6A). While the trend remained the same for nonbrushed leaves, differences were not significant. While similar increases were observed for voltage-gated K^+^ channel (*AKT1*) expression levels in brushed leaves, differences were not significantly (Fig. 6B). When comparing brushed and nonbrushed leaves, expression levels of both *PIP1A* and *AKT1* were generally higher in brushed leaves across all treatments, although these differences were not statistically significant. The exception was *AKT1* in UV-200 plants, where expression in brushed leaves was 3 times higher than in nonbrushed leaves. For all other analysed genes (zeaxanthin epoxidase (*ABA1*), *cytochrome P450 monooxygenase* (*CYP707A4*), Supplementary Fig. S), no significant differences emerged across all treatments.

### Abscisic acid quantification

Abscisic acid (ABA) concentrations were quantified in young leaves with (nonbrushed) and without (brushed) EBCs under all treatment conditions (Supplementary Fig. SC). Salt treatment markedly increased ABA concentrations under both PAR and UV conditions, with values reaching up to a 3.2-fold increase in nonbrushed leaves. A similar trend was observed in brushed leaves, although the increase was not statistically significant. No significant differences were found between brushed and nonbrushed leaves within the same treatment.

## Discussion

### UV-B radiation improved leaf photochemistry of salt-treated plants

While both individual stresses and their combination had limited effects on plant growth, salinity increased the leaf/stem dry weight ratio especially in UV-200 plants (Fig. 1E). Together with the observed decreased shoot elongation under saline conditions, this suggests that resource allocation shifted towards the leaves, likely to sustain transpiration and maintain physiological activity under osmotic stress (Munns and Tester 2008; Jaramillo Roman et al. 2021). An antagonistic interaction occurred between salt and UV-B treatments, with the presence of UV-B improving PSII efficiency while decreasing NPQ in UV-200 plants compared to PAR-200 plants. The substantially decreased NPQ in UV-200 plants was unexpected, as it plays a crucial photoprotective mechanism for dissipating excess energy following excessive radiation absorption (Kromdijk et al. 2022). Nevertheless, this reduced NPQ, combined with similar Φ_PSII_ of UV-200 and control (PAR and UV-treated) plants suggests that the combined stress did not increase photooxidative damage or photoinhibition under our experimental conditions. This improved leaf photochemistry was linked with increases in both chlorophyll and carotenoids concentrations in UV-200 plants, albeit at different extents and thus resulting in a reduced car/chl ratio. As carotenoids are involved in dissipating excess energy and chlorophylls play a central role in absorbing radiation and facilitating electron transport (Guidi et al. 2016; Simkin et al. 2022), this decrease in car/chl ratio of UV-200 plants might explain the simultaneous decline in NPQ declined and improved photosystem efficiency. Nevertheless, the enhanced photochemical capacity in UV-200 plants did not translate into a greater biomass accumulation, which suggests that a greater portion of fixed C was used for stress tolerance mechanisms (e.g. altered ion compartmentation or altered solute transport, as described below). This view is further supported by the lack of photoinhibition or ROS-related damage. By contrast, all measured chlorophyll fluorescence parameters declined in light-adapted PAR-200 leaves. Although total chlorophyll concentration remained unchanged, alterations in chloroplast ultrastructure may have reduced photosynthetic efficiency and energy capture. Although total chlorophyll concentration was not affected, chloroplast ultrastructural alterations may have reduced photosynthetic efficiency or energy capture. These changes, commonly associated with salinity stress, could impair organization and functionality of the photosynthetic apparatus, thereby diminishing photochemical performance (Parida et al. 2003; Shu et al. 2012).

### UV-B improved osmotic adjustment and altered K+ compartmentalization

Combined UV-B and salt treatment improved quinoa water relations. While 200 mm NaCl reduced both RWC and Ѱ_l_ in PAR-treated plants, the combined treatment mitigated these effects, with UV-200 and PAR-0 plants showing comparable values. Similarly, previous studies have shown that UV-B can improve drought tolerance by enhancing leaf hydration, associated with osmolyte accumulation, stomatal closure, and shoot anatomical and morphological modifications (Poulson et al. 2002; Robson et al. 2015; Shoaib et al. 2024). These modifications include increased leaf thickness, increased trichome density, and altered shoot structure, such as plant height and root/shoot ratio. In this study, without root-zone salinity, UV-B and PAR-treated plants maintained similar photosynthetic rates, despite lower stomatal conductance of the former. While decreased *g_s_* was probably caused by ABA increments (Supplementary Fig. S4), attenuated mesophyll limitations could improve CO_2_ diffusion to the chloroplasts of control plants. This hypothesis is supported by SLA data (Supplementary Table S1), with UV-0 plants having a higher SLA than all other treatments. As greater SLA has been associated with thinner leaves and shorter CO₂ diffusion paths that facilitate CO₂ transfer to the chloroplasts (Xu et al. 2013), this may explain high *P_n_* values despite lower *g_s_* of UV-0 plants (Supplementary Fig. S3). As a result, while salt addition to PAR-treated plants approximately halved stomatal conductance, UV plants showed no further declines in *g_s_*. This aligns with previous studies demonstrating that stomatal closure and/or reduced stomatal density decreased stomatal conductance of UV-B-treated plants (Schumaker et al. 1997; Correia et al. 1999; Nogués et al. 1999; Poulson et al. 2002; Reyes et al. 2018; Williams et al. 2022). Additionally, the protective function of UV-B under osmotic stress was associated with greater osmotic adjustment, likely due to increased concentrations of soluble sugars and compatible solutes (Puniran-Hartley et al. 2014).

Foliar osmotic adjustment after salt treatment was primarily driven (82% to 96%) by the accumulation of inorganic ions (K^+^, Na^+^, Cl^−^) rather than organic (fructose, glucose, and sucrose) solutes, with K^+^ playing a dominant role (62% to 77%). This contribution of inorganic solutes was even greater in UV-treated plants, representing a critical energy-saving mechanism. Using abundant inorganic ions is preferable to spending energy to synthesize new organic osmolytes. Thus, salinity and, to a lesser extent, UV decreased leaf sucrose levels compared to PAR-0 plants. As EBCs exhibit low photosynthetic performance, they depend on sugar transporters, like SUCs and SWEETs, for solute transport activity and metabolite production (Kiani-Pouya et al. 2017; Böhm et al. 2018; Bazihizina et al. 2022; Moog et al. 2022). Thus, it could be argued that the decreases in sucrose concentrations and concomitant increases in fructose and glucose in UV, UV-200, and PAR-200 enhanced sucrose breakdown, possibly through the degradative activity of sucrose synthase and/or invertase. This, in turn, would facilitate the breakdown of sucrose into glucose and fructose, providing energy to increase activity in EBCs, either for solute transport activity (e.g. K^+^ movement from EBCs to leaf tissues as discussed below) and/or produce metabolites (e.g. GABA, Kiani-Pouya et al. 2017).

The different salt and UV-B treatments altered foliar K^+^ compartmentalization. Adding salt to the root zone substantially increased shoot K^+^ concentrations, independently of the UV treatment (Moog et al. 2022; Palacios et al. 2024). In particular, the combined salt and UV-B treatment influencing K^+^ allocation between young leaves and the youngest fully expanded leaves, specifically between EBCs and leaf tissues (Fig. 5). When applied individually (PAR-200, UV-0), K^+^ primarily accumulated in EBCs of both young leaves and the youngest fully expanded leaves, as estimated by comparing brushed and nonbrushed leaves. As no significant differences occurred between brushed and nonbrushed leaves in the UV-200 treatment, this suggests either similar K^+^ concentrations between the leaf tissues and EBCs (putative K^+^ relocation from the EBCs to leaf tissues) or a reduced K^+^ accumulation in EBCs, indicating that K^+^ may not have been loaded into the EBCs.

The similar stomatal conductance, ABA concentrations, and expression levels of ABA-related genes in PAR-200 and UV-200 leaves (Supplementary Figs. S3 and S4) likely reflect a salt-induced response independent of the light treatment. While it was not possible to exclude that the improved leaf water relations might be linked with increased ABA levels in UV-200 plants, the improved leaf water relations observed only in this treatment suggest additional mechanisms are involved. In this context, the differential K^+^ compartmentalization between EBCs and leaf tissues in these 2 treatments raises some interesting questions regarding the potential role of K^+^ and the improved water relations in UV-200 plants. Indeed, given the observed differences in K^+^ concentrations and the differential *CqAKT1* expression levels between leaf tissues and EBCs, we hypothesize that the combined UV and salt stress upregulated genes encoding the voltage-gated K⁺ channel and the plasma membrane aquaporin in epidermal cells in UV-200 plants. This would catalyze K⁺ movement from the basal side of EBCs stalk cells into the epidermal cells, thereby creating a K⁺ gradient driving water movement from EBCs to leaf cells. Increased expression of *CqPIP1A* in UV-200 plants would further enhance this process. Overall, EBCs might act as an external reservoir of water for the leaf cells (Shabala and Mackay 2011; Shabala and Pottosin 2014; Shabala et al. 2014).

Adding UV-B radiation did not alter salt-induced changes in Na^+^ and Cl^−^ concentrations or their compartmentalization between EBCs and leaf tissues. Although salt treatment increased these ions by up to 9-fold compared to the values in control plants, their concentrations (particularly Na^+^) were always lower than K^+^ concentrations, as previously observed in salt-treated quinoa (Moog et al. 2022; Palacios et al. 2024). Moreover, leaf Na^+^ and Cl^−^ concentrations were lower than those generally reported for other halophytes and more comparable to those in salt-sensitive glycophytes (e.g. Kim et al. 2021). For instance, in the obligate halophytes *Atriplex mummularia* and *Suaeda dolichostachys* grown with 200 mm NaCl, leaves accumulated 350 to 400 mm Na^+^ (Bazihizina et al. 2009; Katschnig et al. 2013), which is 10 to 20 times higher than the values observed in the present study. Furthermore, most Na^+^ in salt-treated shoots was concentrated in the stems, with concentrations up to 6.8-fold higher than those in young leaves. This therefore explains the relatively low Na^+^ concentrations calculated in EBCs, as only a limited amount of Na^+^ appears to reach the leaf tissues. These results indicate that foliar Na^+^ and Cl^−^ concentrations in quinoa did not reach toxic levels under saline conditions, with their accumulation unlikely to be the primary factor limiting plant growth under our experimental conditions.

### Salt and UV-B effects on secondary metabolism

Rather than uniformly increasing the production of hydroxycinnamic acids with a simple chemical backbone with high UV-B screening efficacy (Table 3, Supplementary Table S7) (Stelzner et al. 2019), UV-B treatment significantly increased the production of a specific hydroxycinnamic acid derivative with peak absorbance at the irradiation wavelength (313 nm). However, salinity minimally affected polyphenol concentrations, with only kaempferol derivatives slightly increasing under single-stress conditions (PAR-200). Although quercetin derivatives with an antioxidant function typically accumulate under osmotic stress in plants (Di Ferdinando et al. 2012; De Souza et al. 2018; Xu et al. 2020), concentrations of these compounds remained remarkably stable across all treatments. Collectively, these results suggest that moderate salinity did not significantly challenge quinoa, as it maintained ionic homeostasis and overall biomass accumulation to some extent.

## Conclusions

This study expands our understanding of halophyte physiological responses to salinity, demonstrating how UV-B radiation and salinity interact to shape plant stress responses and highlighting that investigating the combined effects of these stresses is important to understand the potential agricultural implications. Overall, combined salt and UV-B treatment enhanced the physiological performance of quinoa plants compared to those exposed to salt alone, by increasing photosynthetic efficiency and enhancing water and ion relations. Together, these adaptations mitigated the osmotic component of salinity stress. Understanding whether such interactions modify ion and water relations of different species across the salt tolerance continuum is essential to predict and improve crop performance in salt-affected fields.

## Materials and methods

### Plant material and growth conditions

Quinoa (*Chenopodium quinoa* accession Q20) plants were grown from seeds with universal potting soil composed of neutral sphagnum peat, composted green soil improver, and expanded perlite (less than 5%). The pots were placed in a growth chamber with day/night temperature set at 25 and 22 °C, respectively. The photoperiod was maintained at 12 h/d using time-controlled LED lights (LumiGrow Pro 650), providing an average PAR with an average photon flux density of 210 *μ*mol photons m^−2^ s^−1^.

### Salt and UV-B treatments

After initial measurements confirmed homogeneity of the seedlings, twenty 10-d-old plants were divided into 4 groups (*n* = 5). Each group was assigned to a different treatment to investigate the effect of UV-B radiation, soil salinity, and their interaction. Plants were treated with PAR and tap water (PAR-0), PAR and 200 mm NaCl saline water (PAR-200), UV-B and tap water (UV-0), and UV-B and 200 mm NaCl saline water (UV-200).

UV-B was applied by supplementing PAR for 1 h daily at midday, using 2 tubular Philips UV-B Narrowband PL-L 36 W/01 lamps (Signify NV, Eindhoven, Netherlands), which emit at a peak wavelength of 313 nm. The mean irradiance of the UV-B radiations throughout the experiment was 1.71 W/m^2^, as measured by a PD300-UV Ophir (Ophir Optronics Solutions Ltd., Jerusalem, Israel) radiometer set at 313 nm and previously calibrated with a portable spectroradiometer (model SR9910-PC; Macam Photometrics Ltd., Livingstone, UK) on the used UV-B lamp. To prevent light contamination between treatments, the seedlings treated with PAR and UV-B were placed in 2 separate containers made of UV-blocking LEE 226 plastic film (Lee Filters, Andover, UK) (Supplementary Fig. S5).

### Plant growth

Plants were sampled 26 d after the start of treatments to assess shoot and root fresh and dry mass. Throughout the treatment period, plant growth was monitored weekly by measuring stem and leaf extension with a ruler and the number of leaves on the primary stem. At the end of the experiment, plants were separated into leaves, stems, and roots, and their fresh and dry weights were measured. Using leaf discs collected to estimate leaf RWCs (as described in the section below), we also estimated SLA calculated as the fresh area (cm^2^) divided by dry mass (g).

### Leaf gas exchanges

A LI-COR 6400XT photosynthesis system (Li-6400-40; Li-Cor Inc.), equipped with a LI-6400-40 leaf chamber fluorometer, measured the following parameters: net photosynthetic rate (*P*_n_), stomatal conductance (*g*_s_), intercellular CO_2_ concentration (*C*_i_), maximum quantum efficiency of the PSII (*F*_v_/*F*_m_), capture efficiency of excitation energy by the open (oxidized) PSII reaction center under light (*F*_v_’/*F*_m_’), PSII efficiency in light-adapted leaves (Φ_PSII_), ETR, and NPQ. Measurements (*n* = 5 per treatment) were taken on the youngest fully expanded leaf from 9:00 to 11:30 Am on Day 26. These measurements were conducted at ambient relative humidity, with a reference CO_2_ concentration of 400 *μ*mol mol^−1^, a flow rate of 500 *μ*mol s^−1^, a PAR of 1000 *μ*mol m^−2^ s^−1^, and a leaf chamber temperature set to 25 °C. Chlorophyll fluorescence parameters were measured on both light- and dark-adapted leaves by covering the same leaf with foil for at least 30 min (Netondo et al. 2004; Bazihizina et al. 2016).

### Water relations

Leaf RWC was calculated for each plant (*n* = 5 per treatment) using leaf discs according to the following formula:


$$RWC=\frac{FW-DW}{TW-DW}\times 100$$

where TW stands for turgid weight (measured after 4 h in deionized (DI) water in darkness), FW for fresh weight, and DW for dry weight.

Midday leaf water potential (Ψ_l_; MPa) was measured on 2 leaves per plant (i.e, the second or third pair of youngest fully expanded leaves) using a pressure chamber (Model 1000, PMS, USA) at the end of the experiment. After Ψ_l_ measurements, the leaves were immediately snap-frozen in liquid nitrogen and subsequently used to measure leaf osmotic potential (OP) in the leaf sap. The sap was extracted by placing the thawed leaves in a custom-built separation column and centrifuging at 8000 rpm for 2 min. Leaf sap OP was measured with a psychrometer (PSY1; ICT International, Armidale, NSW, Australia) with relative contributions of the different osmolytes (K^+^, Na^+^, Cl^−^, glucose, fructose, and sucrose, as described in the following paragraphs) calculated using the Van’t Hoff equation with the molar concentration:


π=−RTC

where *R* is the universal gas constant, *T* is the temperature (Kelvin), and *C* is the molar concentration of the solutes (Alarcón et al. 1993; Gori et al. 2023). The calculated OP, based on the sum of each solute OP, closely matched the measured OP (98%). This consistency suggests that the measured ions and soluble carbohydrates were the primary contributors to leaf osmolality.

### Tissue ion concentrations

To better understand ion accumulation in the leaf versus EBCs, ion concentrations (*n* = 5 per treatment) were measured in both nonbrushed and brushed leaves. Hard brushing with a small paintbrush removed the EBCs from the leaves, while nonbrushed leaves retained intact EBCs (Kiani-Pouya et al. 2017; Bazihizina et al. 2022). The 2 youngest fully expanded leaves per plant were sampled, and each was divided into 2 halves along the midrib; one half was brushed to remove the EBCs (brushed leaves), and the other was left intact (nonbrushed leaves). The leaf tissues were then snap-frozen in liquid nitrogen and freeze-dried. Additionally, young leaves were collected as above to have leaves with and without EBCs. These young leaves were immediately frozen and stored at −80 °C until further analysis.

K^+^, Na^+^, and Cl^−^ concentrations were measured in both nonbrushed and brushed leaves, as well as in stems. Ion concentrations in stems and young fully expanded leaves were measured by extracting ground tissues with 0.5 m HNO_3_ as previously described (Bazihizina et al. 2012). In young leaves, ion concentrations were instead measured using the leaf sap (Shabala et al. 2013). The diluted extracts or leaf sap were analyzed for K^+^ and Na^+^ using an atomic absorption spectrophotometer (PinAAcle 500, Perkin Elmer, Waltham, Massachusetts, USA), as previously described (Dainelli et al. 2023). Cl^−^ concentrations were measured using the Sigma Chloride Assay Kit (MAK023, Sigma-Aldrich, St. Louis, MO) according to the manufacturer's protocol. Briefly, 75 *μ*L of reagent was added to 25 *μ*L of sample in a 96-well plate, incubated for 15 min at room temperature, protected from light, and then measured at 620 nm (A620) using a spectrophotometer (Tecan Infinite M200). The reliability of the methods was confirmed by analyzing a reference tissue sample (Rye Grass ERM-CD281, Certified Reference Material) processed through the same procedure.

The K^+^/Na^+^ ratio was calculated for both young leaves and the youngest fully expanded leaves using the ion concentrations from nonbrushed leaves. Additionally, the following formula was used to estimate the ion concentrations within the EBCs:


$$EBCs\;concentration=\frac{NBr-(Br*LW)}{EW}$$

where NBr is the ion concentration in nonbrushed leaves, Br is the ion concentration in brushed leaves, LW is the percentage of weight contributed by the leaf without EBCs, and EW is the percentage of weight contributed by the EBCs in the entire leaf. The EW was determined by weighing leaves before and after the EBCs removal. If the calculated ion concentration in EBCs was negative, the ion concentration was assumed to be 0 mm. Finally, the ion concentration of the youngest fully expanded leaves was used to determine the relative contribution to the leaf OP, as described in the “Water relations” paragraph.

### Pigment quantification

Leaf pigments were also quantified in the nonbrushed youngest fully expanded leaves collected for ion concentration analysis. To determine chlorophyll *a* (Chl *a*), chlorophyll *b* (Chl *b*), and carotenoids (Car), 20 mg of dried and ground leaves were extracted with 1.2 mL of methanol following the method described by Wellburn (1994). After 30 min of extraction in the dark and shaking, the supernatant was measured at 665, 652, and 470 nm using a spectrophotometer (Tecan Infinite M200). The absorbance values were used to calculate the concentrations of Chl *a*, Chl *b*, and Car (*n* = 5 per treatment).

### Sugar quantification

Leaf sap used to measure OP was diluted 2.5-fold with distilled water (*n* = 4 per treatment). A 10 *μ*L aliquot of each sample was injected into a Series 200 HPLC system equipped with a 200-RI detector (PerkinElmer, Bradfrod, CT, USA) and a 7.7 × 300 mm, 8 *μ*m Hi-Plex Ca column (Agilent Technologies, USA) maintained at 85 ± 1 °C, following the method described by Gori et al. (2023). Glucose, fructose, and sucrose were identified by comparing the retention times with those of carbohydrate standards (Sigma-Aldrich, Milano, Italy). Quantification was performed using a 4-point calibration curve for each standard (0.05, 0.1, 0.25, and 0.5 mg/mL) (Supplementary Table S6). The concentrations of soluble sugars (glucose, fructose, and sucrose) were then used to calculate their relative contribution to the leaf OP, as described in the “water relations” paragraph.

### Analysis of polyphenols

One youngest fully expanded leaf per plant was used for water potential measurements and then rapidly snap-frozen for polyphenols analysis (*n* = 5 per treatment). Briefly, polyphenols were extracted from frozen leaves using 60% ethanol for 3 times, as previously described (Sillo et al. 2022). The supernatants from the samples were partitioned and defatted using *n*-hexane to remove chlorophylls and other substances that could interfere with chromatographic analysis. The hydroethanolic phase was then dried using a Concentrator plus (Eppendorf, Italy), and the residue was redissolved in a MeOH: Milli-Q water solution (1:1 v/v, pH 2.5 adjusted with formic acid). Polyphenol separation and quantification were performed using a Perkin Elmer Flexar liquid chromatography system (Perkin Elmer, Bradford, CT, USA), equipped with a quaternary 200Q/410 pump and an LC 200 diode array detector. The resuspended samples were injected into an Agilent Zorbax C18 analytical column (250 mm 4.6 mm, 5 m), maintained at 30 °C, to achieve separation and quantification of the polyphenols. The mobile phase consisted of (A) Milli-Q water and (B) acetonitrile, both acidified with 0.1% formic acid. The flow rate was set to 0.4 mL min^−1^, using the following gradient program: 0 to 1 min: 3% B, 1 to 55 min: 40% B, 55 to 60 min: 40% B, and 60 to 61 min: 3% B. A 10-min conditioning step was used to return to the initial conditions. Chromatograms were recorded at 280 and 350 nm, while spectral data from all peaks were collected over a wavelength range of 210 to 590 nm. Polyphenols were identified by comparing the UV–vis spectral characteristics and retention times with those of authentic standards and data from the literature (Paśko et al. 2008; Gawlik-Dziki et al. 2013; Vázquez-Luna et al. 2019; Al-Qabba et al. 2020). Quantification of the peaks was performed using calibration curves prepared with the following standards: gallic acid, caffeic acid, kaempferol-3-O-glucoside, rutin, and apigenin-7-O-glucoside (all from SigmaAldrich—Merck KGaA, Darmstadt, Germany). Polyphenols were extracted from fresh leaves, and their content was calculated as milligrams per gram of dry weight by normalizing the data based on the leaf water content.

### Gene expression analysis

Using the available transcriptome of brushed and nonbrushed quinoa leaves (Bazihizina et al. 2022), 5 genes expressed in quinoa leaves and EBCs and linked to water and ion transport and ABA regulation were selected: (A) *AKT1*, voltage-gated K^+^ channel, (B) *PIP1A*, plasma membrane aquaporin, (C) *ABA1*, zeaxanthin epoxidase involved in the first step of ABA biosynthesis, and (D) *CqCYP707A4,* cytochrome P450 monooxygenase encoding ABA 8′-hydroxylase. After 26 d of treatments, 2 young leaves per plant were harvested, 1 was brushed, and the other one was left intact, and then immediately frozen in liquid nitrogen and kept at −80 °C for further analysis. Subsequently, total RNA was extracted using the Plant/Fungi Total RNA Purification Kit (Norgen Biotek Corp) according to the manufacturer's protocol from 50 mg of leaf tissue ground in liquid nitrogen. On-column DNase treatment was assessed using Norgen's RNase-Free DNase I Kit (Norgen Biotek Corp). Electrophoresis using 1% agarose gel was performed for all RNA samples to check for RNA integrity, followed by spectrophotometric quantification. RNA was then reverse transcribed using SuperScript IV Reverse Transcriptase kit (Life Technologies, UK) with oligo(dT)20 primers. Gene expression analysis was performed using the CFX Connect real-time PCR detection system (Bio-Rad, Hercules, CA, USA) employing 30 ng of cDNA for each reaction and SsoAdvanced Universal SYBR Green Supermix (Bio-Rad), according to the manufacturer's instructions for the detection system (Bio-Rad). Elongation factor 1-alpha (*EF1alpha*) was used as a housekeeping gene, and 3 technical replicates were performed for each biological replicate (*n* = 3). Primers were designed by using Primer3 software (http://primer3.ut.ee/) and double-checked using net primer software (http://www.premierbiosoft.com/netprimer/), except for the housekeeping primers (Böhm et al. 2018). A complete list of primers used for real-time quantitative PCR (RT-qPCR) analyses is given in Supplementary Table S8. Relative gene expression levels were calculated according to Livark and Schmittgen (2001).

### ABA quantification

Leaves were freeze-dried and ground into powder. Samples (approximately 20 mg dry weight) were mixed with deionized water (1:50 extraction ratio) and shaken at 4 °C overnight to extract ABA. After centrifuging the extracts at 15,000 rpm for 5 min, the ABA concentration of the supernatant was directly measured via radioimmunoassay using the monoclonal antibody AFRC MAC 252 (Quarrie et al. 1988).

### Data analysis

All statistical analyses were performed using GraphPad Prism 9.5.1 for Windows (GraphPad Prism Inc., San Diego, CA, USA). The data were assessed for normal distribution through a Shapiro–Wilk test and homogeneity distribution of variance through Bartlett's test, before a 2-way ANOVA. Additionally, a 3-way ANOVA was performed to examine potential interactions between salt, light, and tissue ion concentration. Treatment differences (*P*-value ≤ 0.05) were identified using Tukey's multiple comparison test.

### Accession numbers

Sequence data from this article can be found in the GenBank/EMBL data libraries under accession numbers listed in Supplementary Table S9.

## Supplementary Material

kiaf569_Supplementary_Data

## Data Availability

The data underlying this article will be shared on reasonable request to the corresponding author.

## Supplementary data

The following materials are available in the online version of this article.

**Supplementary Figure S1.** Overview of quinoa plant morphology and epidermal bladder cells (EBCs) structures.

**Supplementary Figure S2.** Leaf elongation (cm) in quinoa under the 4 treatments.

**Supplementary Figure S3.** Responses of leaf gas exchange parameters of quinoa plants to the 4 treatments on Day 26.

**Supplementary Figure S4.** Gene expression patterns of *CqABA1* and *CqCYP707A4* (cytochrome P450 monooxygenase) and ABA levels in nonbrushed (i.e. intact) and brushed young leaves of quinoa after 26 d of treatment.

**Supplementary Figure S5.** The experiment set up for the study.

**Supplementary Table S1.** Dry weight of leaves, stem, and roots, shoot/root ratio and specific leaf area (SLA) of the youngest fully expanded leaves of quinoa after 26 d with the 4 treatments.

**Supplementary Table S2.** Maximum quantum efficiency of photosystem II (*F*_v_/*F*_m_) in quinoa after 26 d under 4 treatments.

**Supplementary Table S3.** 3-way ANOVA results of K^+^, Na^+^, and Cl^−^ concentrations across tissue type, radiation treatment, and salt treatment.

**Supplementary Table S4.** K^+^/Na^+^ ratio in quinoa young leaves (YL) and youngest fully expanded leaves (YFEL).

**Supplementary Table S5.** Concentration of Na⁺ and Cl⁻ (mM) in nonbrushed (N) and brushed (B) young leaves (YL) and youngest fully expanded leaves (YFEL).

**Supplementary Table S6.** Concentration of soluble sugars in the sap of youngest fully expanded leaves.

**Supplementary Table S7.** Secondary metabolite concentration in the youngest fully expanded leaves of quinoa after 26 d of treatment.

**Supplementary Table S8.** List of primer pairs used for qPCR analysis of gene expression.

**Supplementary Table S9.** AGI identifiers and corresponding NCBI accession numbers (NM for mRNA transcripts and NP for proteins) of the closest *Arabidopsis thaliana* homologous genes used for RT-qPCR analysis of gene expression.

## Dive Curated Terms

The following phenotypic, genotypic, and functional terms are of significance to the work described in this paper:

AKT1 Gramene: AT2G26650
AKT1 Araport: AT2G26650

PIP1A Gramene: AT3G61430
PIP1A Araport: AT3G61430

ABA1 Gramene: AT5G67030
ABA1 Araport: AT5G67030

CYP707A4 Gramene: AT3G19270
CYP707A4 Araport: AT3G19270

water CHEBI: CHEBI:15377

chlorophyll CHEBI: CHEBI:28966

RWC Gramene: Relative Water Content
RWC Araport: Relative Water Content
