# Supplementary material for: Ultraviolet B radiation improves salt-induced responses in the facultative halophyte Chenopodium quinoa
Source: Plant Physiol. 2025 Nov 10;200(3):kiaf569. doi: 10.1093/plphys/kiaf569 (PMC13017555; doi:10.1093/plphys/kiaf569)
Supplement: kiaf569_Supplementary_Data [file kiaf569_supplementary_data.zip › Supplementary data OK.pdf]

SUPPLEMENTARY DATA

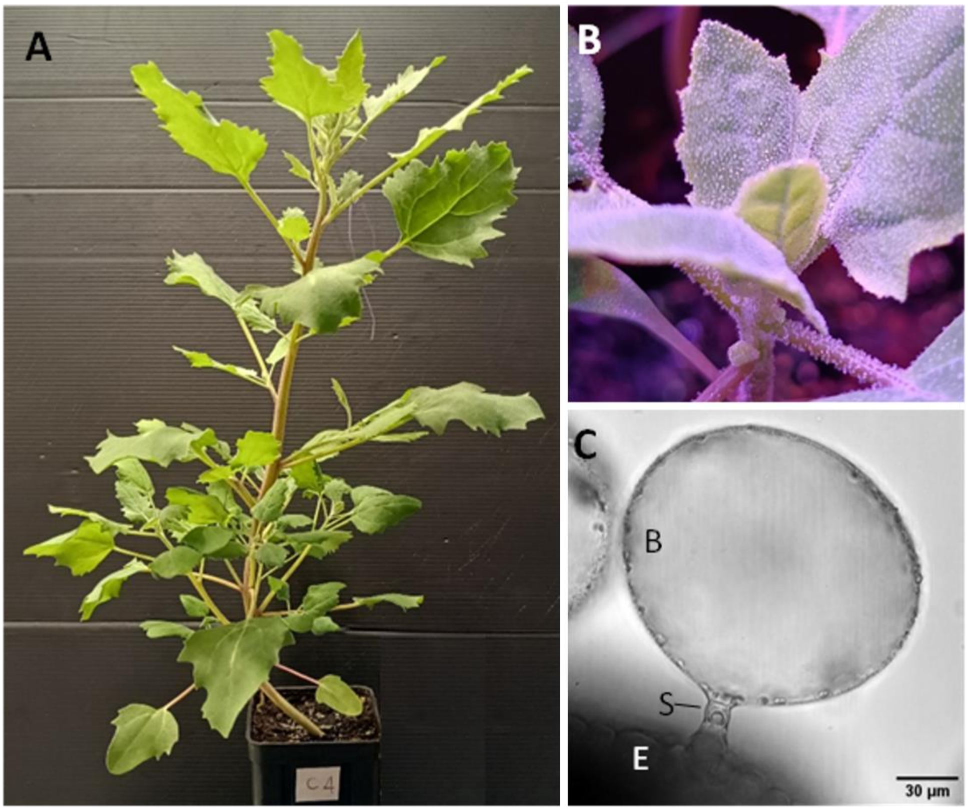

Supplementary Figure S1. Overview of quinoa plant morphology and epidermal bladder cells (EBCs) structures. (A) 36-day-old quinoa plants, (B) detail of a quinoa seedling, with surface EBCs visible as small white dots on the leaves and petioles, (C) Image of EBCs from a control plant (image used also in the Figure 5) showing key structures: B - bladder, S -stalk cell, E - epidermal cells.

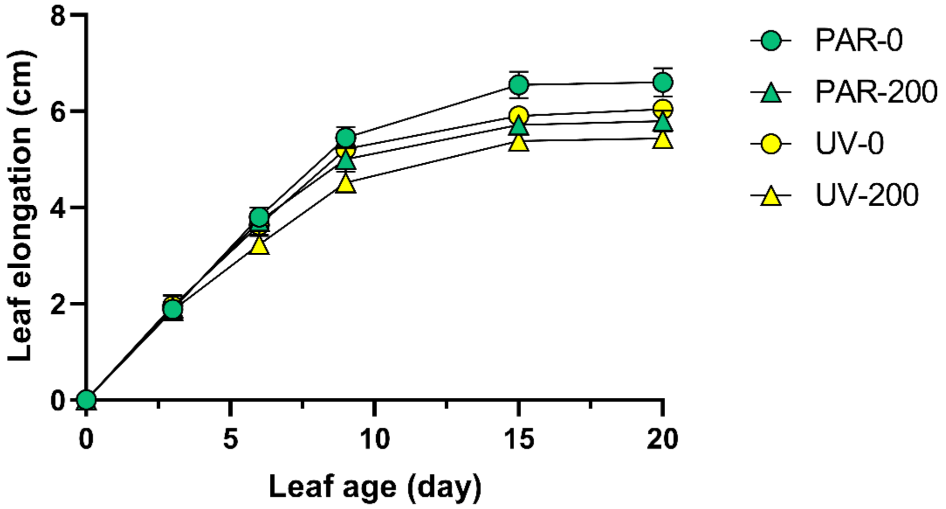

Supplementary Figure S2. Leaf elongation (cm) in quinoa under the four treatments. No statistically significant differences were detected among treatments ($p > 0.05$, two-way ANOVA followed by Tukey's test).

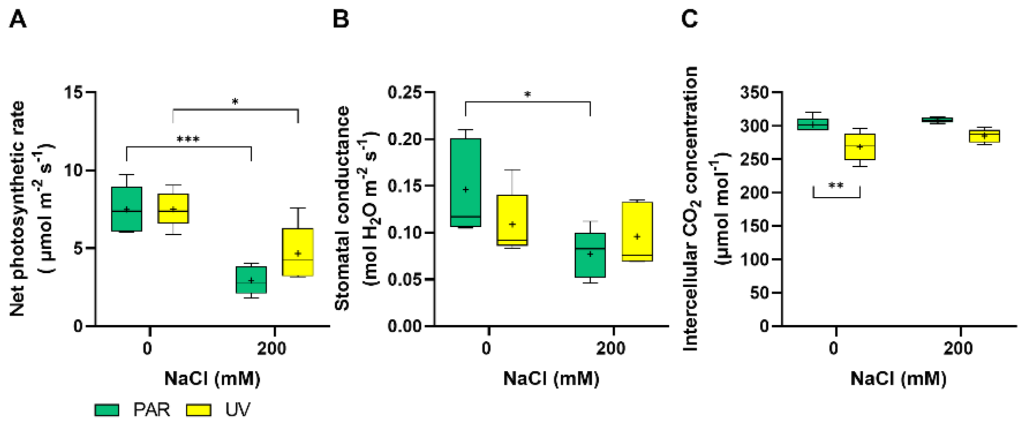

Supplementary Fig. S3. Responses of leaf gas exchange parameters of quinoa plants to the four treatments on day 26. (A) P_n (net photosynthetic rate), (B) g_s (stomatal conductance), and (C) C_i (intercellular CO_2). Asterisks (* $p \leq 0.05$, ** $p \leq 0.01$, *** $p \leq 0.001$, **** $p \leq 0.0001$) indicate significant differences based on a two-way ANOVA followed by Tukey's multiple comparison test. The top and bottom of each box represent the 25th and 75th percentiles, the horizontal line inside each box represents the median, the "+" symbol indicates the average (n=5), and the whiskers show the minimum and maximum values.

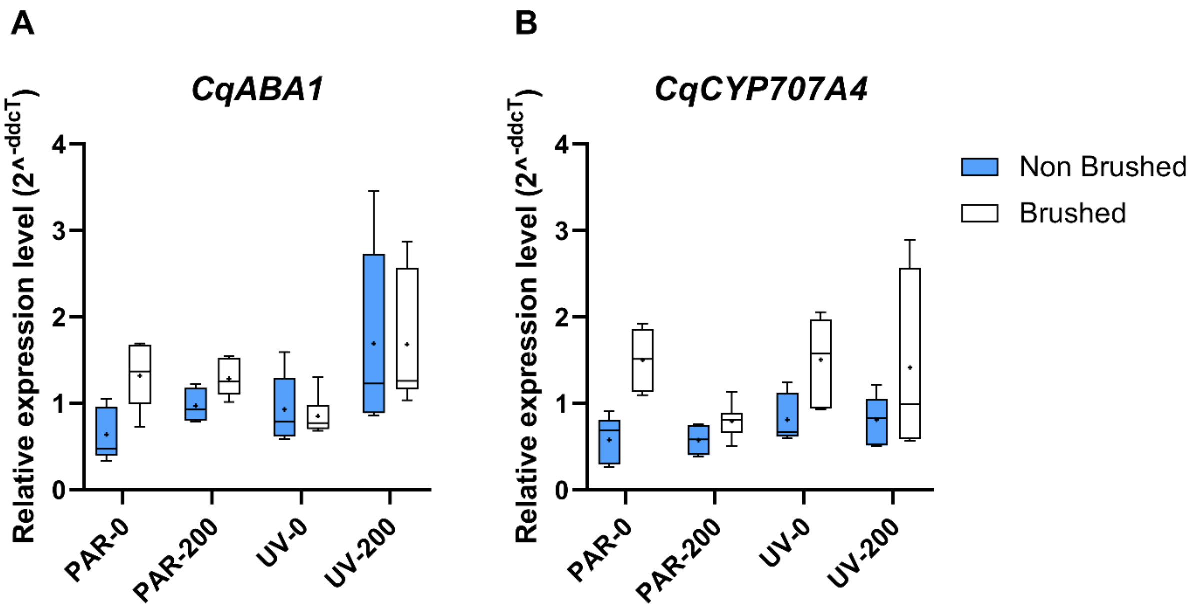

C

ABA (ng g^{-1} DW)	PAR-0	UV-0	PAR-200	UV-200
Non brushed	264 ± 63 a	336 ± 56 a	840 ± 188 b	778 ± 73 b
Brushed	261 ± 67	269 ± 8	610 ± 77	598 ± 31

Supplementary Fig S4. Gene expression patterns of *CqABA1* and *CqCYP707A4* (cytochrome P450 monooxygenase) and ABA levels in non-brushed (i.e. intact) and brushed young leaves of quinoa after 26 days of treatment. (A) *ABA1*, encoding for zeaxanthin epoxidase, which is involved in generating the epoxycarotenoid precursor of the ABA biosynthetic pathway (B) *CqCYP707A4,* cytochrome P450 monooxygenase encoding ABA 8′-hydroxylase, the key step in the oxidative catabolism of ABA, and (C) ABA leaf content. In (A) and (B) no significant differences were found among treatments ($p > 0.05$, two-way ANOVA followed by Tukey's test). The x-axis of the boxplot represents the light and salt treatment. The top and bottom of each box represent the 25th and 75th percentiles, respectively. The horizontal line inside each box represents the median, the «+» inside each box represents the average, and the whiskers represent the minimum and maximum values (n=5). In (C) table shows, where present, significant differences ($p \leq 0.05$, two way ANOVA followed by Tukey's test) between salt treatments (PAR-0 vs. PAR-200, and UV-0 vs. UV-200) using lowercase letters, and between radiation treatments (PAR-0 vs. to UV-0, and PAR-200 vs. UV-200) using an asterisk. Data are presented as means ± SE (n=3).

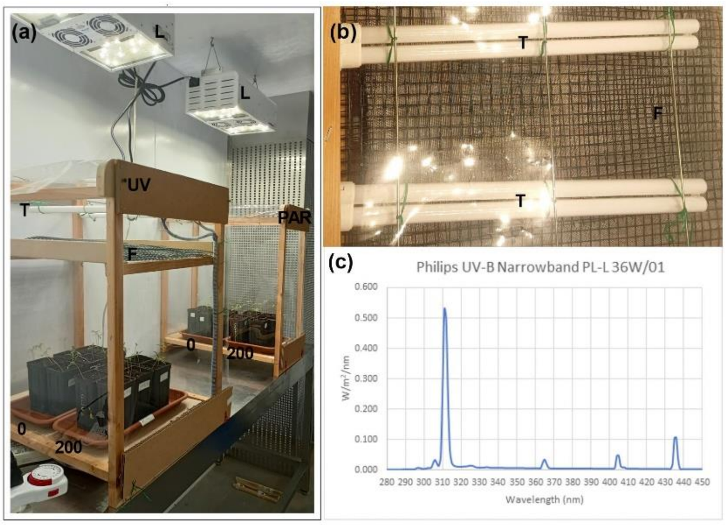

Supplementary Figure S5. The experiment set up for the study. (a) Division of plants into two custom-built chambers for the different radiation and salt treatments. The "**UV**" chamber, designated for UV-treated plants, measures 40cm in width, 50cm in length, and 60cm in height. "**T**" represents the UV-B lamp, and "**F**" indicates the physical filters placed under the lamp to reduce the irradiance. The "**PAR**" chamber, designated for control plants, measures 30 cm in width, 50 cm in length, and 60 cm in height. In each chamber, plants are further divided into two groups: "**0**" for tap water irrigation, and "**200**" for irrigation with 200mM saline water. The PAR lamps are labelled "**L**" above the two chambers. (b) Detail of the UV-B tubular lamps and physical filters. (c) Emission spectrum of the UV-B lamp, with a main peak is at 313 nm.

Supplementary Table S1. Dry weight of leaves, stem, and roots, shoot/root ratio and specific leaf area (SLA) of the youngest fully expanded leaves of quinoa after 26 days with the four treatments. The table shows only statistically significant differences ($p \leq 0.05$) between salt treatments (PAR-0 vs. PAR-200, and UV-0 vs. UV-200) using lowercase letter. Data are presented as means ± SE (n=5 for leaves and stem, and n=3 for roots and shoot/root).

	PAR-0	UV-0	PAR-200	UV-200
Leaves (g)	1.25 ± 0.12 *a*	1.20 ± 0.06 *a*	1.26 ± 0.14 *a*	1.06 ± 0.05 *a*
Stem (g)	0.72 ± 0.03 *a*	0.65 ± 0.04 *a*	0.40 ± 0.05 *b*	0.33 ± 0.02 *b*
Roots (g)	0.92 ± 0.16 *a*	0.70 ± 0.030 *a*	0.47 ± 0.031 *b*	0.41 ± 0.040 *a*
Shoot/Root ratio	2.41 ± 0.13 *a*	2.45 ± 0.07 *a*	3.87 ± 0.31 *b*	3.28 ± 0.29 *a*
SLA (cm^2 g^{-1})	469.24 ± 15.34	550.50 ± 40.74 *a*	447.88 ± 11.86	443.51 ± 14.27 *b*

Supplementary Table S2. Maximum quantum efficiency of photosystem II (F_v/F_m) in quinoa after 26 days under four treatments. No statistically significant differences were detected among treatments ($p > 0.05$, two-way ANOVA followed by Tukey's test). Data are means ± SE (n = 5).

	PAR-0	UV-0	PAR-200	UV-200
F_v/F_m	0.81 ± 0.004	0.82 ± 0.008	0.82 ± 0.004	0.81 ± 0.006

Supplementary Table S3. Three-way ANOVA results of K^+, Na^+, and Cl^- concentrations across tissue type, radiation treatment, and salt treatment. Significant *p*-values have been highlighted in bold.

K^+	Source of Variation	% of total variation	*p*-value
	Tissue	1.217	0.0718
	Light	0.4985	0.1375
	Salt	82.11	**<0.0001**
	Tissue x Light	0.6986	0.2127
	Tissue x Salt	1.394	**0.05**
	Light x Salt	2.268	**0.0023**
	Tissue x Light x Salt	1.331	0.0569
Na^+	Tissue	28.05	**<0.0001**
	Light	0.1262	0.3284
	Salt	45.58	**<0.0001**
	Tissue x Light	1.894	**0.0017**
	Tissue x Salt	16.16	**<0.0001**
	Light x Salt	0.2779	0.1494
	Tissue x Light x Salt	1.701	**0.003**
Cl^-	Tissue	14.65	**<0.0001**
	Light	0.1219	0.4212
	Salt	74.08	**<0.0001**
	Tissue x Light	0.2696	0.4881
	Tissue x Salt	0.7428	0.1456
	Light x Salt	0.6091	0.076
	Tissue x Light x Salt	0.6368	0.19

Supplementary Table S4. K^+/Na^+ ratio in quinoa young leaves (YL) and youngest fully expanded leaves (YFEL). The ratio was calculated using the concentration from non-brushed leaves. The table shows only statistically significant differences ($p \leq 0.05$) between salt treatments (PAR-0 vs. PAR-200, and UV-0 vs. UV-200) using lowercase letters, and between light treatments (*PAR-0* vs. UV-0, and *PAR-200* vs. UV-200) using an asterisk. Data are presented as means ± SE (n=5).

K^+/Na^+			
PAR-0	UV-0	PAR-200	UV-200

YL	162.7 ± 27.7 *a**	94.1 ± 10.6 *a**	26.9 ± 4.8 *b*	23.2 ± 2.3 *b*
YFEL	42.2 ± 5.3 *a*	39.2 ± 5.6 *a*	10.2 ± 1.5 *b*	7.7 ± 1.1 *b*

Supplementary Table S5. Concentration of Na^+ and Cl^- (mM) in non-brushed (N) and brushed (B) young leaves (YL) and youngest fully expanded leaves (YFEL). No statistically significant differences were detected between non-brushed and brushed leaves for either Na^+ or Cl^- ($p > 0.05$, two-way ANOVA followed by Tukey's test). Data are presented as means ± SE (n = 5).

mM			PAR-0	UV-0	PAR-200	UV-200
Na^+	YL	N	1.7 ± 0.3	3.5 ± 0.5	14.7 ± 3.4	15.2 ± 2.6
		B	1.8 ± 0.2	4.0 ± 0.5	18.0 ± 3.2	18.0 ± 2.1
	YFEL	N	6.8 ± 1.0	8.5 ± 1.0	48.2 ± 4.0	62.2 ± 5.8
		B	6.6 ± 0.6	10.3 ± 1.0	47.9 ± 10.5	64.7 ± 9.9
Cl^-	YL	N	102.4 ± 2.8	79.7 ± 4.6	243.7 ± 44.5	261.5 ± 12.4
		B	85.5 ± 4.8	77.0 ± 3.7	231.5 ± 34.8	272.0 ± 11.1
	YFEL	N	34.4 ± 1.7	30.3 ± 1.9	164.1 ± 18.4	157.1 ± 8.0
		B	34.6 ± 3.3	31.3 ± 2.4	163.5 ± 10.8	158.7 ± 5.9

Supplementary Table S6. Concentration of soluble sugars in the sap of youngest fully expanded leaves. The table shows significant differences ($p \leq 0.05$) between salt treatments (PAR-0 vs. PAR-200, and UV-0 vs. UV-200) using lowercase letters, and between radiation treatments (PAR-0 vs. to UV-0, and PAR-200 vs. UV-200) using an asterisk. Data are presented as means ± SE (n=4).

mM	PAR-0	UV-0	PAR-200	UV-200
Sucrose	47.4 ± 5.1 *a**	15.5 ± 3.2 *a*	27.3 ± 3.7 *b*	13.6 ± 2.8 *a*
Glucose	14.0 ± 1.4 *a*	18.1 ± 1.2 *a*	23.4 ± 2.3 *b*	16.8 ± 1.3 *a*
Fructose	6.1 ± 1.3 *a*	18.4 ± 2.4 *a*	21.4 ± 0.65 *b*	11.2 ± 2.1 *a*

Supplementary Table S7. Secondary metabolite concentration in the youngest fully expanded leaves of quinoa after 26 days of treatment. The table shows only statistically significant differences ($p \leq 0.05$) between salt treatments (PAR-0 vs. PAR-200, and UV-0 vs. UV-200) using lowercase letters, and between radiation treatments (PAR-0 vs. UV-0, and PAR-200 vs. UV-200) using an asterisk.

Peak	Compounds (mg/g DW)	Rt	λmax	PAR-0	UV-0	PAR-200	UV-200
1	Gallic acid	28.34	280	0.07 ± 0.008 a	0.04 ± 0.006	0.15 ± 0.03 b	0.08 ± 0.02
2	Sinapic acid derivative	34.37	326	0.18 ± 0.05 a	n.d	0.72 ± 0.23 b	n.d
3	Sinapic acid derivative	36.2	324	0.16 ± 0.02 *	0.55 ± 0.04 *	0.17 ± 0.02 *	0.43 ± 0.02 *
4	Sinapic acid derivative	37.12	320	2.93 ± 0.22 *	1.16 ± 0.11 *	2.37 ± 0.32 *	0.74 ± 0.07 *
5	Sinapic acid derivative	39.25	326	0.46 ± 0.02 *	0.27 ± 0.04 *	0.51 ± 0.02 *	0.25 ± 0.04 *
6	Quercetin-3-rutioside	40.31	350	4.64 ± 0.68	5.73 ± 0.45	4.17 ± 0.71	4.44 ± 0.77
7	Rutin	43.18	350	4.00 ± 0.65	3.89 ± 1.20	3.34 ± 0.02	2.55 ± 0.41
7a	Kaempferol	43.18	350	n.q	0.85 ± 0.17	1.76 ± 0.25	1.40 ± 0.09
8	Coumaric acid derivative	43.89	311	4.77 ± 0.98	5.79 ± 0.21	3.37 ± 0.34	4.52 ± 0.31
9	Coumaric acid derivative	44.88	311	0.26 ± 0.05 *	7.78 ± 0.42 a*	0.28 ± 0.04 A	5.64 ± 0.44 b*
10	Coumaric acid derivative	45.72	326	1.67 ± 0.30	1.19 ± 0.13	1.82 ± 0.25	1.86 ± 0.27
11	Karmpferol-3-glucoside	46.3	351	0.34 ± 0.08	0.34 ± 0.06	0.30 ± 0.12	0.44 ± 0.07
12	Putative apigenin	49.2	334	0.89 ± 0.11	0.57 ± 0.07	0.80 ± 0.13 *	0.33 ± 0.05 *

Supplementary Table S8: List of primer pairs used for RT-qPCR analysis of gene expression.

Protein	Gene	AGI code[a]	Quinoa gene[b]	Forward	Reverse Primer
**ABA**	***ABA1***	***At5G6703***	TRINITY_GG_30513_c11_	5'-TGTTTGGGCATACAGACGCT -3'	5'-CCTGCACCCACATCTGATGA-3'
**Cytochrom**	***CYP707A***	***At3G1927***	TRINITY_GG_30513_c12_	5'-GCAAGGAAGAGGTTGGGACA-3'	5'-CCAAGAAGGTCCTTTGTTGCC-3'

**PIN-**	***PIN5***	***At5G1653***	TRINITY_GG_17810_c2_g	5'-AAAGCTGGGACTGGCACTTC-3'	5'-GTCAACTTTGGTCCACACGC-3'
**Plasma**	***PIP1A***	***At3G6143***	TRINITY_GG_15985_c38_	5'-GCCAACTTGTGATGTGCTCG-3'	5'-GCAGGCCCAATACATCTTGC-3'
**K+**	***AKT1***	***At2G2665***	TRINITY_GG_5307_c17_g	5'-TGGTGCTGATCCTGATTGCA-3'	5'-AAGTTTCACCACCGCCTCAT-3'
**Uv**	***UVR8***	***At5G6386***	TRINITY_GG_14241_c37_	5'-GCATGGAAGGATCTGGTGGT-3'	5'-ACCATTGTCCCTTGAGCCTG-3'
**Elongation**	***EF1α***	***At1g0794***	AUR62027945	5'- GTACGCATGGGTGCTTGACAAACT	5'- ATCAGCCTGGGAGGTACCAGTAA

[a] *Identifiers correspond to the AGI code of the closest Arabidopsis thaliana homologous gene identified by blast search.*

[b] *Original sequences (TRINITY_GG) from RNA-seq data deposited in the ArrayExpress database at EMBL-EBI (http://www.ebi.ac.uk/arrayexpress). Stalk cell study: Array Express ID: E-MTAB-10363*

*** from Böhm et al. 2018*

Supplementary Table S9: AGI identifiers and corresponding NCBI accession numbers (NM for mRNA transcripts and NP for proteins) of the closest *Arabidopsis thaliana* homologous genes used for RT-qPCR analysis of gene expression.

Protein function	Gene name	AGI code[a]	NCBI code-transcripts	NCBI code-protein
**ABA**	***ABA1***	***At5G67030***	NM_180954.3	NP_851285.1
**Cytochrome**	***CYP707A4***	***At3G19270***	NM_112814.2	NP_566628.1
**PIN-**	***PIN5***	***At5G16530***	NM_121659.5	NP_197157.4
**Plasma**	***PIP1A***	***At3G61430***	NM_116008.4	NP_191702.1
**K+**	***AKT1***	***At2G26650***	NM_128222.6	NP_180233.1
**Uv**	***UVR8***	***At5G63860***	NM_125781.4	NP_201191.1
**Elongation**	***EF1α***	***At1g07940***	NM_100668.3	NP_563801.1'
