# Supplementary figures and images for: Ultraviolet B radiation improves salt-induced responses in the facultative halophyte Chenopodium quinoa
Source: Plant Physiol. 2025 Nov 10;200(3):kiaf569. doi: 10.1093/plphys/kiaf569 (PMC13017555; doi:10.1093/plphys/kiaf569)

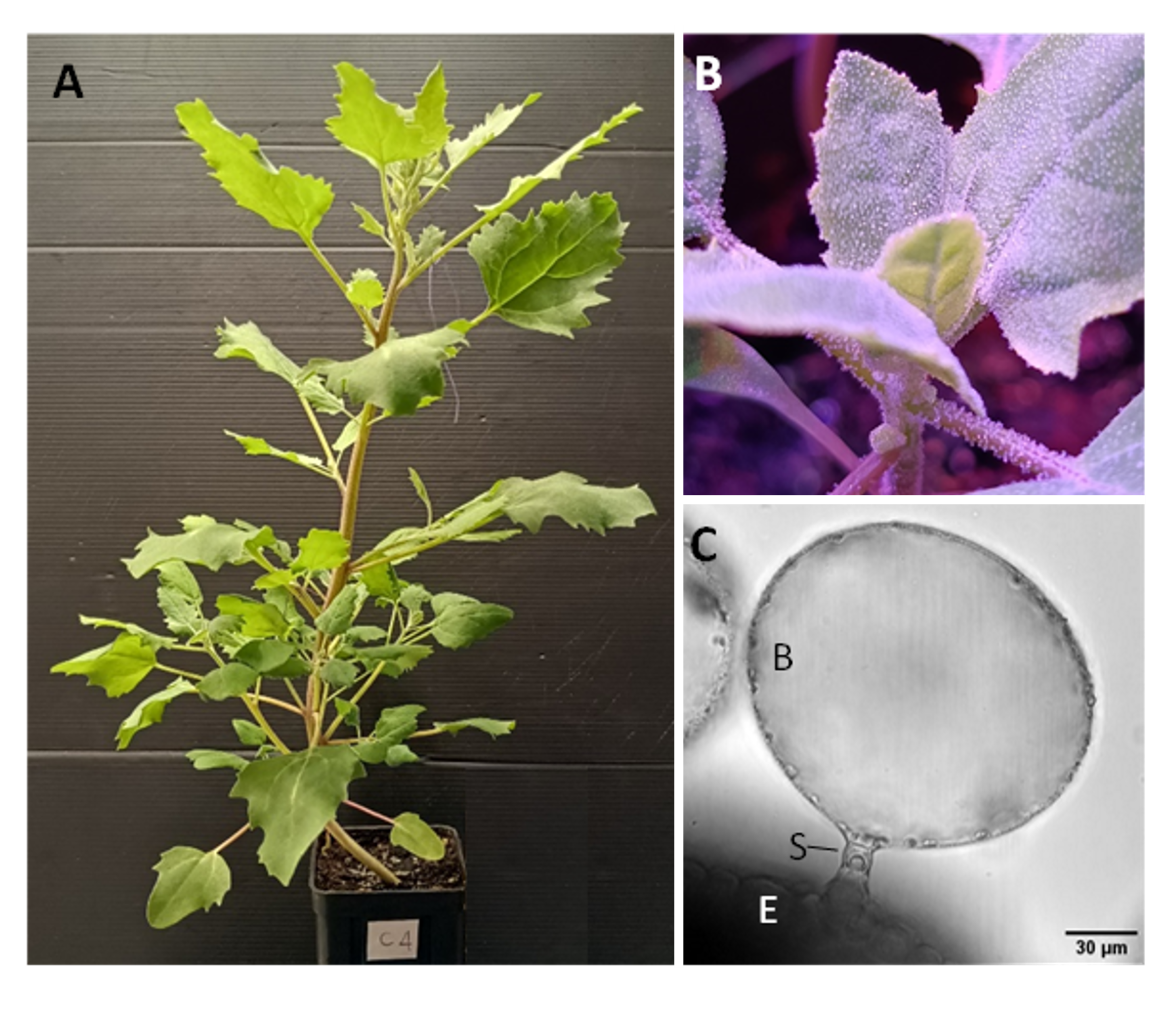

Supplement: kiaf569_Supplementary_Data [file kiaf569_supplementary_data.zip › Supplementary FIG S1.tif]

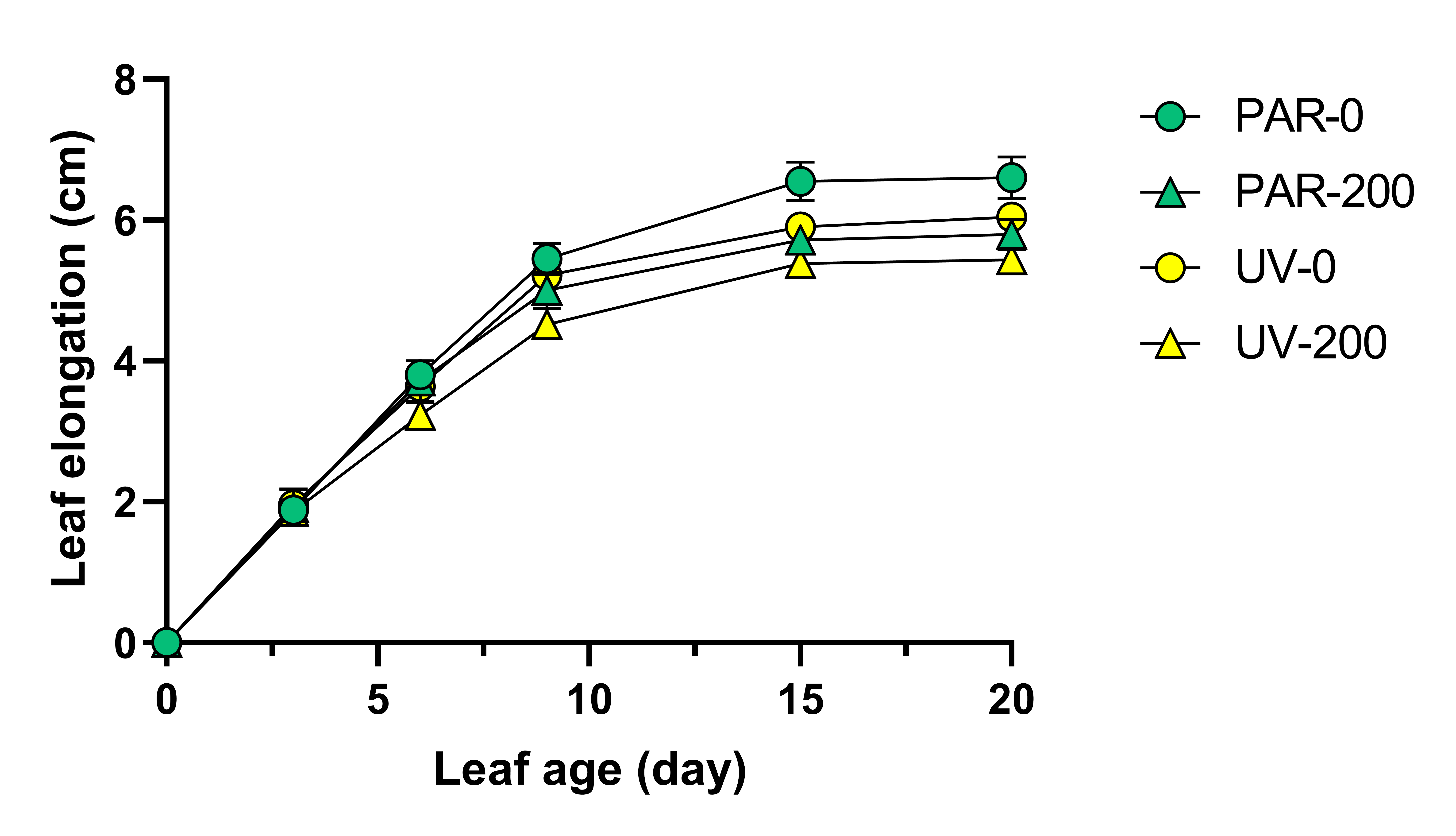

Supplement: kiaf569_Supplementary_Data [file kiaf569_supplementary_data.zip › Supplementary FIG S2.tif]

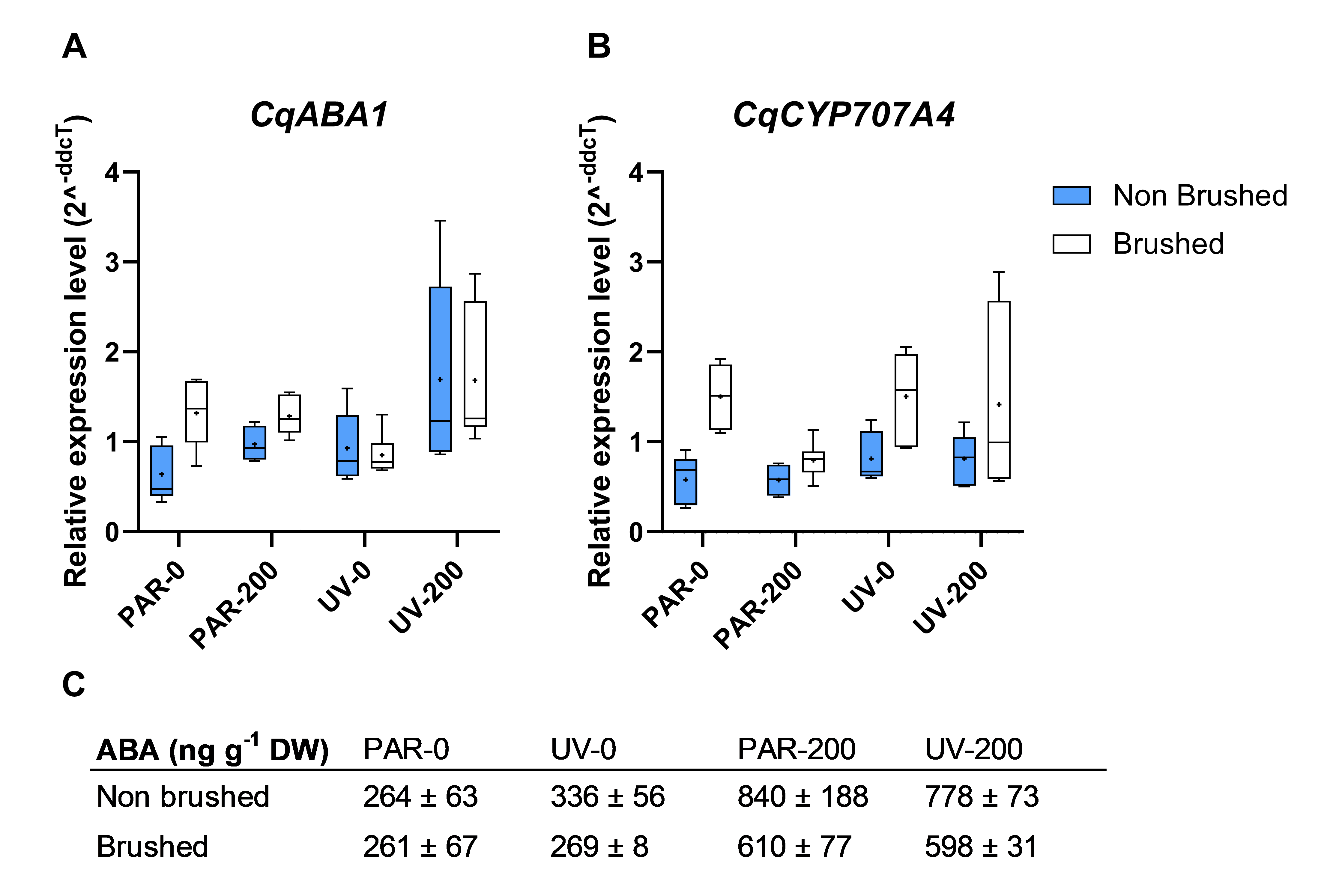

Supplement: kiaf569_Supplementary_Data [file kiaf569_supplementary_data.zip › Supplementary_FIG_S4.tif]

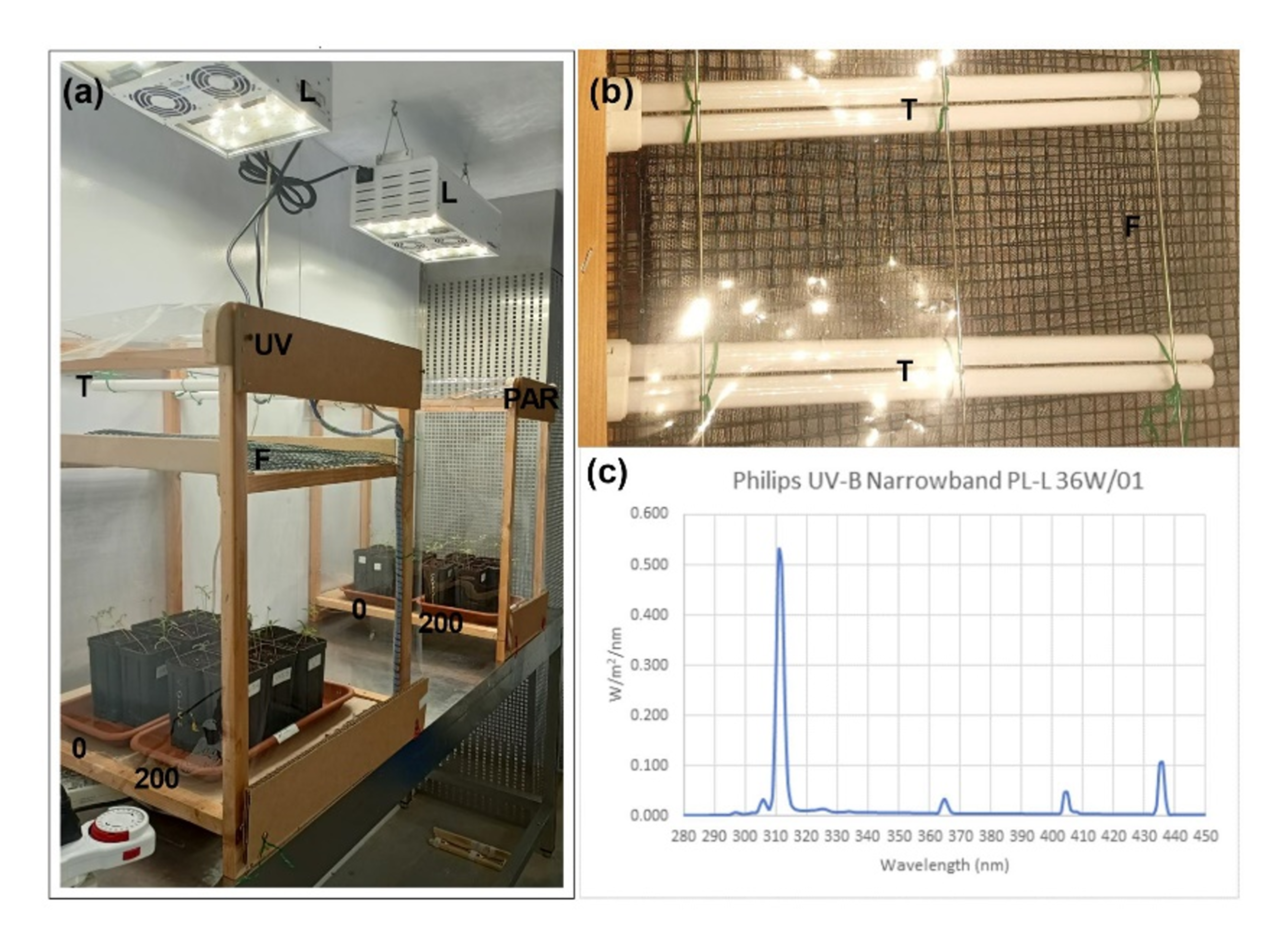

Supplement: kiaf569_Supplementary_Data [file kiaf569_supplementary_data.zip › Supplementary_FIG_S5.tif]

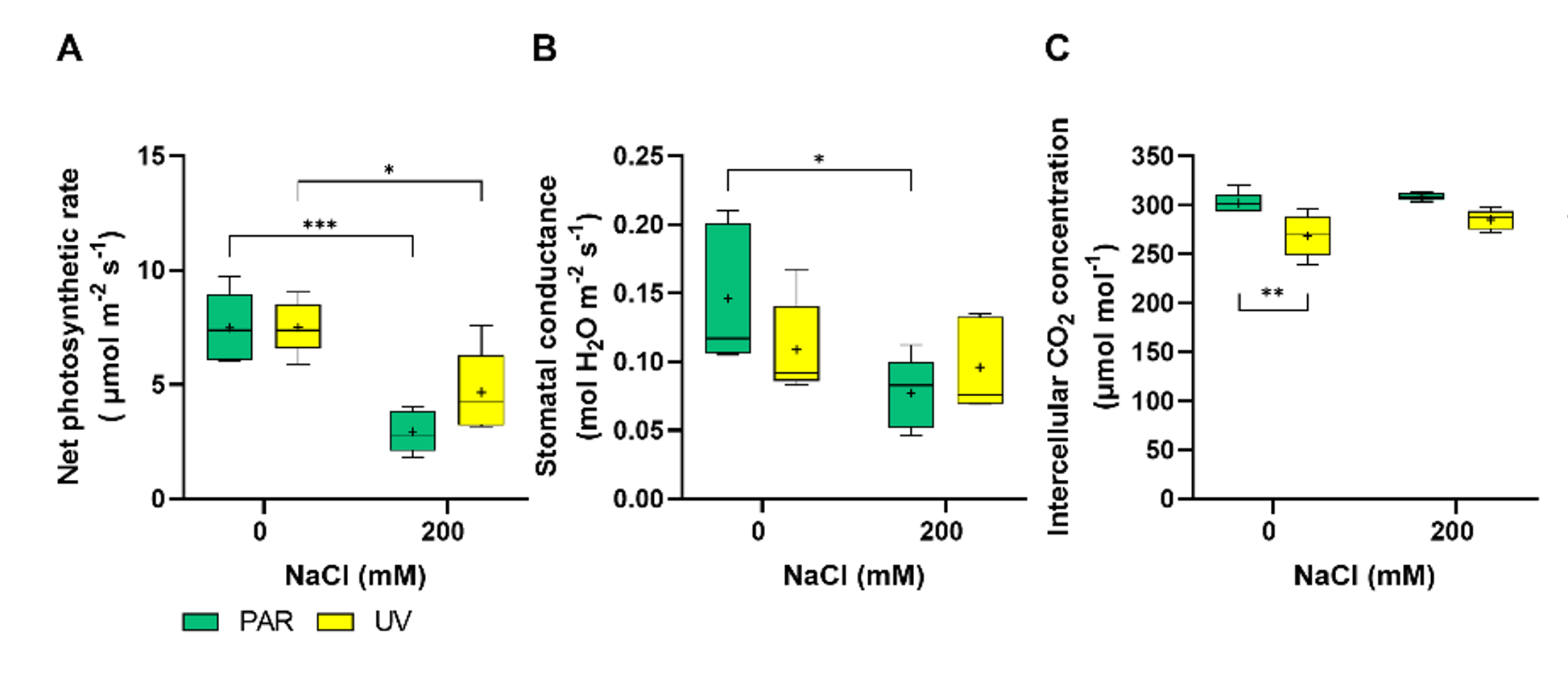

Supplement: kiaf569_Supplementary_Data [file kiaf569_supplementary_data.zip › Supplemetary_FIG_S3.tif]
